# The endoplasmic reticulum pool of Bcl-xL prevents cell death through IP3R-dependent calcium release

**DOI:** 10.1038/s41420-024-02112-1

**Published:** 2024-08-01

**Authors:** Rudy Gadet, Lea Jabbour, Trang Thi Minh Nguyen, Olivier Lohez, Ivan Mikaelian, Philippe Gonzalo, Tomas Luyten, Mounira Chalabi-Dchar, Anne Wierinckx, Olivier Marcillat, Geert Bultynck, Ruth Rimokh, Nikolay Popgeorgiev, Germain Gillet

**Affiliations:** 1 Université de Lyon, Université Claude Bernard Lyon 1, INSERM 1052, CNRS 5286, Centre Léon Bérard, Centre de recherche en cancérologie de Lyon, 28 rue Laennec, 69008 Lyon, France; 2 Laboratoire de Biochimie, CHU de Saint-Etienne, Université Jean Monnet, Saint-Étienne, France; 3 ProfilXpert. Faculté de Médecine Lyon Est, 8 rue Guillaume Paradin, 69008 Lyon, France; 4 Institut Universitaire de France, Paris, France; 5 Hospices civils de Lyon, Laboratoire d’anatomie et cytologie pathologiques, Centre Hospitalier Lyon Sud, chemin du Grand Revoyet, 69495 Pierre Bénite, France; 6 Present Address: KU Leuven. Laboratory Molecular and Cellular Signaling. Department Cellular and Molecular Medicine., Campus Gasthuisberg O/N-I bus 802 Herestraat 49, BE-3000 Leuven, Belgium

**Keywords:** Cancer, Apoptosis

## Abstract

Apoptosis plays a role in cell homeostasis in both normal development and disease. Bcl-xL, a member of the Bcl-2 family of proteins, regulates the intrinsic mitochondrial pathway of apoptosis. It is overexpressed in several cancers. Bcl-xL has a dual subcellular localisation and is found at the mitochondria as well as the endoplasmic reticulum (ER). However, the biological significance of its ER localisation is unclear. In order to decipher the functional contributions of the mitochondrial and reticular pools of Bcl-xL, we generated genetically modified mice expressing exclusively Bcl-xL at the ER, referred to as ER-xL, or the mitochondria, referred to as Mt-xL. By performing cell death assays, we demonstrated that ER-xL MEFs show increased vulnerability to apoptotic stimuli but are more resistant to ER stress. Furthermore, ER-xL MEFs displayed reduced 1,4,5-inositol trisphosphate receptor (IP3R)-mediated ER calcium release downstream of Phospholipase C activation. Collectively, our data indicate that upon ER stress, Bcl-xL negatively regulates IP3R-mediated calcium flux from the ER, which prevents ER calcium depletion and maintains the UPR and subsequent cell death in check. This work reveals a moonlighting function of Bcl-xL at the level of the ER, in addition to its well-known role in regulating apoptosis through the mitochondria.

## Introduction

Bcl-xL is an anti-apoptotic member of the Bcl-2 family of apoptosis regulators [1]. This family is composed of numerous proteins that are classified, based on their structure and function. Multidomain Bcl-2 homologs, which comprise cell death inhibitors, such as Bcl-2 and Bcl-xL, and executioners, such as Bax and Bak, possess up to four homology domains referred to as Bcl-2 homology (BH1-4) domains. BH3-only proteins (Bad, Bid, Puma…), which only regroup death accelerators, are also considered as Bcl-2 homologs, even though being from different phylogenic origins [2]. Activation of the proapoptotic multi BH domain- containing Bax/Bak induce mitochondrial outer membrane permeabilization (MOMP), a key checkpoint upstream of the activation of the caspase cascade resulting in apoptosis [3, 4].

Dysregulations affecting the network of Bcl-2 homologs might have detrimental outcomes, such as auto-immune or cancer diseases. Indeed, Bcl-xL is upregulated in hepatocellular and renal carcinoma [5, 6], as well as pancreatic cancer [7]. Furthermore, Bcl-xL was shown to play a role in the invasion of human malignant glioma [8] and colorectal cancer cells [9]. However, in breast cancer, there is evidence that Bcl-xL does not affect tumor growth but increases metastatic potential, independent from apoptosis [10, 11].

Throughout the years, non-canonical functions of the Bcl-2 family of proteins have been progressively unmasked. Indeed, besides their mitochondrial localization, most of the Bcl-2 proteins translocate to other subcellular compartments where they perform diverse functions regarding cell cycle, metabolism and signal transduction [12]. A number of these functions are carried out through intracellular calcium (Ca^2+^) fluxes [13]. The endoplasmic reticulum (ER) is a welcoming hub for a number of Bcl-2 proteins where they interact with ER-localised Ca^2+^ channels and impinge on Ca^2+^ homeostasis regulation. For instance, Bcl-2, *via* its BH4 domain, interacts with the modulatory and transducing region and ligand-binding region of the inositol trisphosphate receptor 1 (IP3R1) thus inhibiting ER Ca^2+^ discharge [14, 15]. In contrast, Bcl-xL can interact with the C-terminal IP3R1 tail, thereby sensitizing the channel to a low concentration of IP3 [16]. Hence, this interaction induces ER Ca^2+^ release and promotes Ca^2+^ oscillations required for mitochondrial energetics [17]. In addition to this, Bcl-xL can also interact with the coupling domain of IP3R1, thereby exerting an inhibitory impact on the channel [16, 18]. Of note, Bcl-xL that is exclusively targeted to the ER was reported to be capable of restoring Ca^2+^ homeostasis in Bcl-xL-deficient mouse embryonic fibroblasts (MEFs), even though the underlying mechanisms are not fully elaborated [19]. In fact, although Bcl-xL has been reported to contribute to major processes governing cancer progression [5, 11] and embryonic development [20], the respective contributions of Bcl-xL subcellular pools in these processes remain unclear.

To address this matter, we generated genetically modified mice by Cre-LoxP recombination system in which Bcl-xL is targeted exclusively either to the ER (ER-xL mice) or to the mitochondria (Mt-xL mice). Both recombinant mice survive the embryonic stage, however, contrary to Mt-xL individuals which apparently did not show a specific phenotype in normal conditions, ER-xL mice died shortly after birth [21]. Upon cytotoxic drug treatment, MEFs derived from ER-xL mice were found to be sensitive to Staurosporine, an inducer of the mitochondrial pathway of apoptosis, but, interestingly, resisted to ER stress inducers such as Thapsigargin and Tunicamycin. We thus hypothesized that Bcl-xL might confer this resistance through its influence on ER Ca^2+^ release. Indeed, in this study, we show that, in stressful conditions, ER-targeted Bcl-xL decreases ER Ca^2+^ release through IP3R, compared to mitochondria-targeted Bcl-xL. Altogether, our data shed the light on a moonlighting function of Bcl-xL at the ER where it dampens IP3R-dependent Ca^2+^ release, acting thus as an inhibitor of the ER stress response.

## Results

### Bcl-xL silencing sensitizes to inducers of the unfolded protein response

**Fig. 1:**
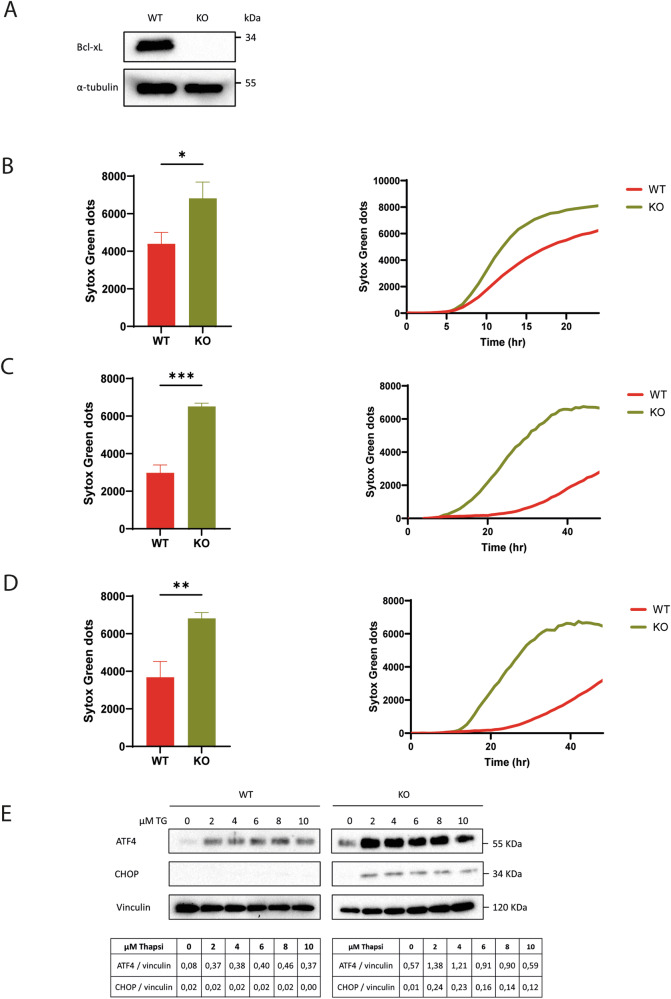
Bcl-xL protects from ER stress. **A** Western blotting analysis of Bcl-xL endogenous levels in WT and *bclx* KO MEFs. α-Tubulin was used as a loading control. **B–D** Cell death quantification in WT and *bclx* KO MEFs treated with 1 µM Staurosporine for 16 h (**B**), 10 µM Thapsigargin for 48 h (**C**) or 2.5 µM Tunicamycin for 48 h (**D**). The difference between treated cells and negative controls (DMSO-treated) is shown on the vertical axis of each panel (Sytox GREEN^TM^ dots). Left panels: histograms showing the results from three independent experiments. A Student’s t test was performed with N_WT_ = 3, N_KO_ = 3 (mean ± SD; *, *p* < 0.05; **p < 0.01; ***p < 0.001). Right panels: representative kinetics. **E**. Western blotting analysis of the expression of ATF4 & CHOP, used as UPR markers, in the presence of increasing amounts of Thapsigargin (TG) for 24 h in WT and *bclx* KO MEFs. Vinculin was used as a loading control.

Bcl-xL is an anti-apoptotic Bcl-2 family member known to repress Bax-dependent MOMP. Indeed, *bclx*-knockout (KO) MEFs were found more sensitive to the protein kinase C inhibitor Staurosporine, an activator of the mitochondria-dependent apoptosis pathway, as shown using Sytox Green^TM^- based cell death assay (Fig. 1A, B). In the course of this study, we also observed that these KO cells were more sensitive to Ca^2+^ stress-inducing agents such as the sarco/endoplasmic reticulum Ca^2+^ ATPase (SERCA) inhibitor Thapsigargin and the GlcNAc phosphotransferase inhibitor Tunicamycin (Fig. 1C, D). These observations were confirmed using Caspase 3 assays (Supplementary Fig. 1). Thus, in addition to mitochondria-dependent apoptosis, *bclx* silencing may have consequences regarding ER-dependent processes. Indeed, Thapsigargin is a potent ER stress activator that signals through the unfolded protein response (UPR) suggesting a role of Bcl-xL in the ER stress response. Accordingly, we investigated the influence of Bcl-xL on the UPR by analyzing the expression profile of typical UPR markers [22]. In an interesting manner, CHOP and ATF4 levels were increased in KO MEFs upon Thapsigargin treatment, compared to WT MEFs (Fig. 1E), supporting that Bcl-xL may dampen the cellular response to ER stress inducers. The fact that ATF4 levels were spontaneously increased in KO MEFs in the absence of thapsigargin (Fig. 1E) confirmed that these cells were to some extent, prone to ER stress.

### Generation of single subcellular localisation mutants of Bcl-xL

The above data suggest that Bcl-xL is a negative regulator of ER stress, and might thus physically interact with the ER. We therefore analyzed Bcl-xL subcellular localisation in MEFs isolated from C57BL/6 mice embryos. Subcellular fractionation experiments on WT MEFs highlighted the multiple subcellular localizations of Bcl-xL at the mitochondria, the cytosol, and the ER (Supplementary Fig. 2A).

**Fig. 2:**
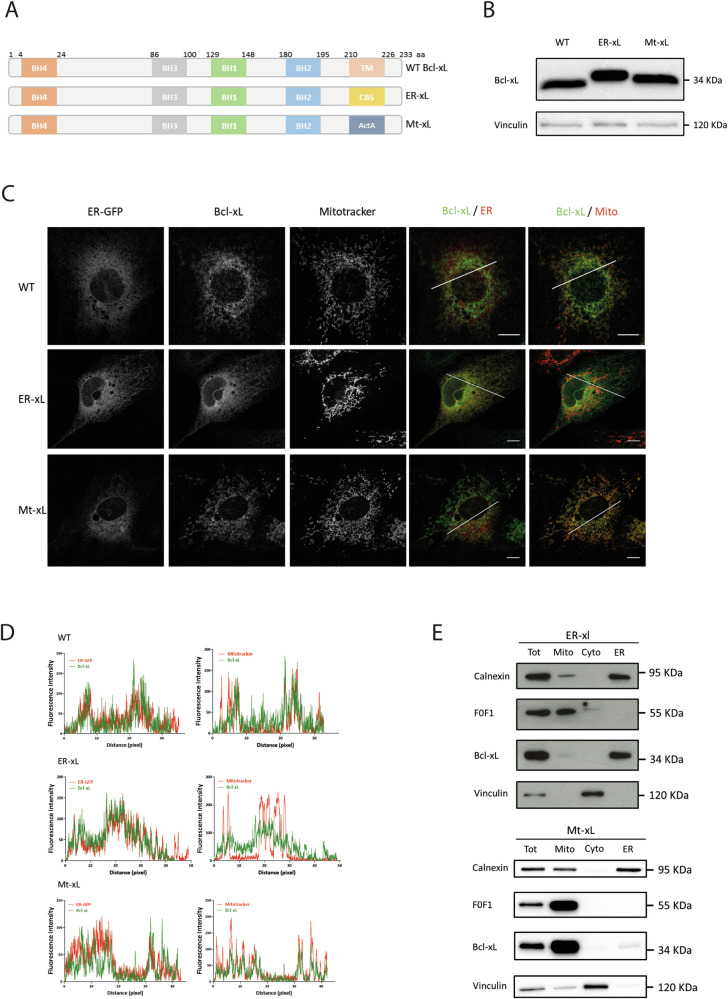
Caracterization of ER-xL and Mt-xL MEFs. **A** Diagram of Bcl-xL products in WT, ER-xL and Mt-xL MEFs. These cells respectively express Bcl-xL with intact TM domain, CB5 targeting sequence and ActA targeting sequence. MEFs were obtained from E13 embryos. Generation of recombinant mice is described in [21]. **B** Western blotting. Detection of Bcl-xL in WT, ER-xL and Mt-xL MEFs. Vinculin was used as loading control. **C** Immunofluorescence. Bcl-xL subcellular localisation in WT, ER-xL and Mt-xL MEFs. Co-localisation was assessed using ER-EGFP transfection and Mitotracker staining for ER and mitochondrial localization, respectively. Scale bar: 10 µm. **D** Profile plots of fluorescence signals. Fluorescence intensity along the white segments on merged images (last two right panels, see “**C**” above) were quantified by ImageJ software. **E** Western blotting. Detection of Bcl-xL in subcellular fractions from ER-xL MEFs (top panels) and Mt-xL MEFs (lower panels). (Tot) whole-cell lysates; (Mito) mitochondria; (Cyto) cytosol; (ER) endoplasmic reticulum. Vinculin was used as a cytosol marker, Calnexin as an ER marker and F0F1 ATPase as a mitochondrial marker.

In order to decipher the functional contribution of the ER and mitochondrial pools of Bcl-xL, we generated recombinant C57BL/6 mice by the Cre-LoxP system in which Bcl-xL is exclusively at the ER (ER-xL) or at the mitochondria (Mt-xL). An engineered vector containing the WT Bcl-xL exon 3 and Cytochrome B (CB5) or ActA mutant sequences that respectively target proteins to the ER or the mitochondria, was inserted to replace the WT *bclx* exon 3 in floxed mice. Cre-dependent recombination resulted in WT exon 3 excision and CB5 or ActA mutant expression, see [21]. Accordingly, three protein products could be generated: WT Bcl-xL with intact transmembrane (TM) domain, ER-xL with CB5 targeting sequence, and Mt-xL with ActA targeting sequence (Fig. 2A).

Recombinant products were validated at both protein and genomic levels using western blot and polymerase chain reaction (PCR), respectively (Fig. 2B & Supplementary Fig. 2B). Bcl-xL protein expression was checked in MEFs derived from WT, ER-xL and Mt-xL embryos at embryonic day E13. It should be noted that ER-xL (245 aa) and Mt-xL (242 aa) are slightly longer than WT Bcl-xL (233 aa), as shown in Fig. 2B.

We next aimed at verifying the subcellular localisation of Bcl-xL in MEFs immortalized by serial passaging. To do so, we performed immunofluorescence experiments on all three types of MEFs transfected with ER-targeted EGFP. Bcl-xL was found at the ER in ER-xL MEFs, at the mitochondria in Mt-xL MEFs and co-localised in both organelles in WT MEFs, as indicated by the profile plots of fluorescence intensity (Fig. 2C, D). Subcellular localisation of Bcl-xL was confirmed by fractionation (Fig. 2E) and ImmunoGold labelling (Supplementary Fig. 2C, D). Quantification of protein levels at the ER revealed that the amount of ER-resident Bcl-xL was approximately three times higher in ER-MEFs, compared to WT-MEFs (Supplementary Fig. 2E).

### Bcl-xL at the ER, and not at the mitochondria, confers resistance to Ca^2+^ stress

**Fig. 3:**
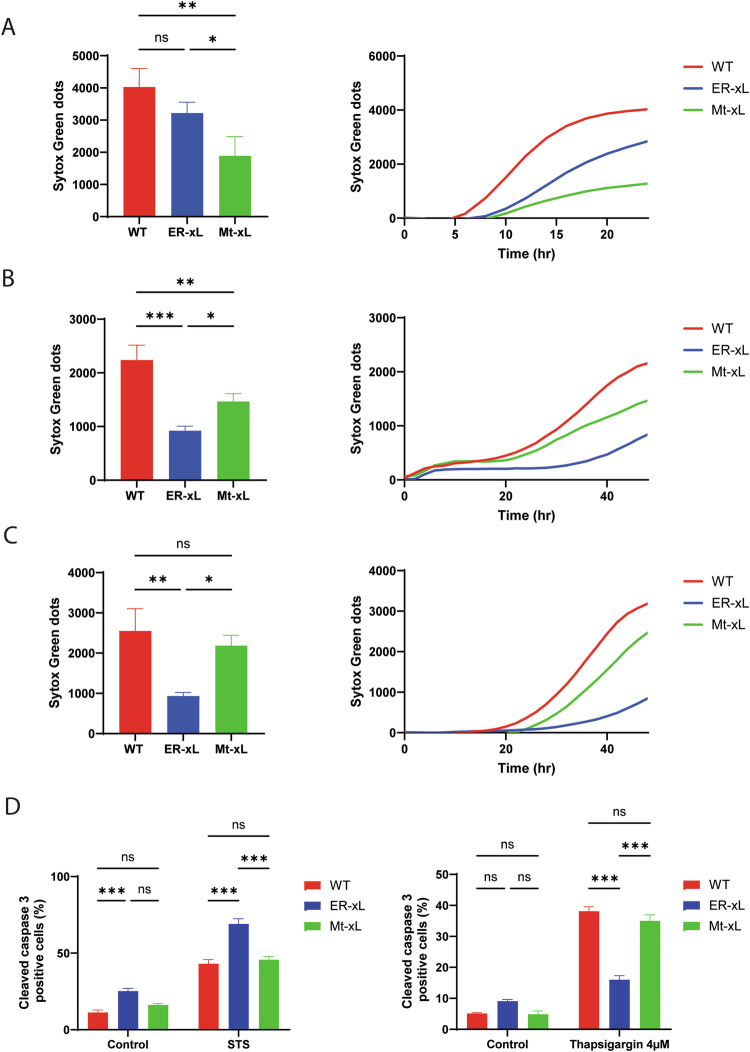
ER-xL MEFs resist Ca^2+^-dependent cell death. **A**–**C** Cell death quantification (Sytox GREEN^TM^-positive cells) in WT, ER-xL and Mt-xL MEFs treated with 1 µM Staurosporine (**A**), 10 µM Thapsigargin (**B**) or 2.5 µM Tunicamycin (**C**). The difference between treated cells and negative controls (DMSO-treated) is shown on the vertical axis of each panel (Sytox GREEN^TM^ dots). Left panels: histograms showing the results from three independent experiments. A one way ANOVA test was performed accordingly with N_WT_ = 3, N_ER-xL_ = 3, N_Mt-xL_ = 3 (mean ± SD; ns, non-statistically significant, *p* > 0.05; **, *p* < 0.01; ***, *p* < 0.001). Cell death was measured at 24 h for Staurosporine and at 48 h for Thapsigargin and Tunicamycin. Right panels: representative kinetics. **D** Detection of cleaved Caspase 3 through fluorescence microscope analysis in WT, ER-xL and Mt-xL MEFs after treatment with 250 nM Staurosporine (STS) for 6 h. Cells treated with DMSO were used as negative controls. Histograms show cleaved Caspase 3 quantification. A two-way ANOVA was performed with N_WT Control_ = 16, N_ER-xL control_ = 17, N_Mt-xL control_ = 17, N_WT STS_ = 18 N_ER-xL STS_ = 18, N_Mt-xL STS_ = 18. (mean ± SEM; ns, non-statistically significant, ***, p < 0.001). E. Detection of cleaved Caspase 3 through fluorescence microscope analysis in WT, ER-xL and Mt-xL MEFs after treatment with 4 µM Thapsigargin (TG) for 24 h. Cells treated with DMSO were used as negative controls. Histograms (left panel) show cleaved Caspase 3 quantification. A two-way ANOVA was performed with N_WT_ = 15, N_KO_ = 15, N_WT_ = 15, N_KO_ = 15, N_WT_ = 15, N_KO_ = 15. (mean ± SEM; ns, non-statistically significant, ***, *p* < 0.001).

Having verified the expression and localisation of Bcl-xL, we wanted to test the anti-apoptotic potential of the protein by treating the MEFs with cell death inducers and measuring cell death counting Sytox Green^TM^-positive cells. Actually, Mt-xL MEFS appeared to better resist to STS treatment, compared to ER-xL MEFs (Fig. 3A). Fluorescence-activated cell sorting (FACS) measurements showed subG1 population increase in ER-xL MEFs, suggesting also higher susceptibility to cell death (Supplementary Fig. 3A). In contrast, ER-xL MEFs were found to better resist to the Ca^2+^ stress inducers Thapsigargin and Tunicamycin, compared to Mt-xL MEFs and WT MEFs (Fig. 3B, C). These observations were confirmed by measuring caspase 3 activation (Fig. 3D & Supplementary Fig. 3B, C).

Collectively, these data indicate that ER-xL MEFs are more resistant to Ca^2+^ stress inducers than Mt-xL MEFs, while being able to activate the intrinsic mitochondria-dependent apoptosis pathway to a comparable extent, with respect to WT. In this regard, it should be noted that ER-xL MEFS exhibit a significantly higher rate of spontaneous cell death compared to WT (Fig. 3D, left panel), suggesting that ER-xL MEFS are more susceptible to apoptosis, presumably due to lack of mitochondrial Bcl-xL.

### ER-based Bcl-xL inhibits IP3R-Ca^2+^ release

Mechanistically, we hypothesized that Bcl-xL influence could be through ER Ca^2+^ regulation. Indeed, Bcl-xL presumably acts as a potent modulator of Ca^2+^ fluxes at the ER as it was previously reported to physically interacts with BH3 domain-like motifs in the IP3R C-terminal tail through its hydrophobic pocket. Yet, Bcl-xL was also shown to perform a biphasic regulation of IP3R gating at the ER, whereby low Bcl-xL protein levels sensitized IP3Rs while high Bcl-xL protein levels inhibited IP3Rs [17, 23, 24].

**Fig. 4:**
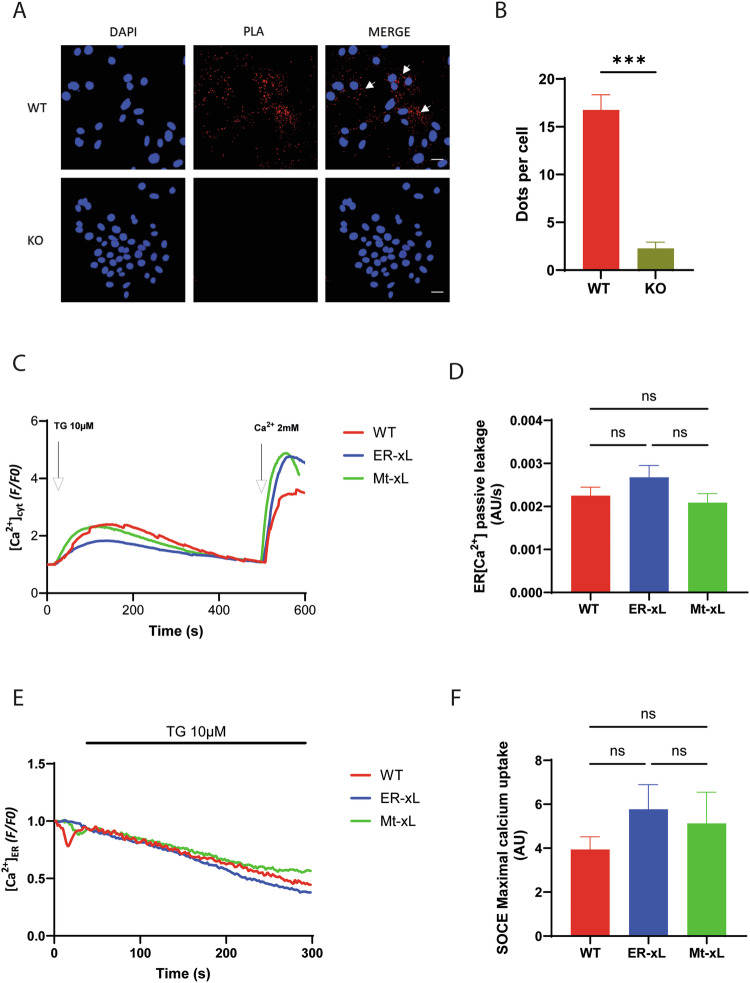
Bcl-xL at the ER affects intracellular Ca^2+^ fluxes. **A** Representative images showing the endogenous interactions between IP3R - C terminus and Bcl-xL in WT and KO MEFs through a Proximity Ligation Assay (PLA). DAPI was used to stain nuclei (blue dots). Red dots correspond to PLA-positive signals, indicating close proximity between Bcl-xL and IP3R (middle upper panel and right upper panel). The middle bottom panel (PLA/KO, negative control) is unstained, confirming the specificity of the labelling observed in the top panel (PLA/WT). White arrows point to red dots. Scale bar: 30 µm. **B** Quantification of PLA experiments. A Mann Whitney’s test was performed with N_WT_ = 30, N_KO_ = 31 (mean ± SEM; ***, *p* < 0.001). **C** Representative curves of ER passive Ca^2+^ leakage in WT, ER-xL, and Mt-xL MEFs transfected with CEPIA-1er after 10 μM Thapsigargin injection. **D** Quantification of the slope coefficient of ER passive calcium leakage in MEFs. A one way ANOVA was performed with N_WT_ = 14, N_ER-xL_ = 16, N_Mt-xL_ = 17 (mean ± SEM; ns, non-statistically significant). **E**. Representative curves of Store Operated Calcium Entry (SOCE) assessed with 5 μM Fluoforte after draining the ER from calcium by 10 μM Thapsigargin injection, followed by 2 mM CaCl_2_ injection, in WT, ER-xL and Mt-xL MEFs. **F** The ratio of fluorescence (F/F0) indicating the maximal calcium uptake after CaCl_2_ injection is shown. A one way ANOVA test was performed with N_WT_ = 3, N_ER-xL_ = 3, N_Mt-xL_ = 3 (mean ± SD; ns, non-statistically significant).

First, we demonstrated that Bcl-xL-IP3R interaction actually existed in WT MEFs. Indeed, Proximity Ligation Assay (PLA) using antibodies targeting specifically Bcl-xL and IP3R detected a significantly higher number of PLA dots in WT MEFs (16.76%) compared to KO cells (2.28%) (Fig. 4A, B).

Furthermore, we evaluated the effect of Bcl-xL on passive ER Ca^2+^ leak from the ER Ca^2+^ stores in ER-xL and Mt-xL MEFs. To this end, ER Ca^2+^ passive leakage (in the absence of IP3 stimulation) was analyzed after Thapsigargin treatment in MEFs transfected with R-CEPIA1er. In fact, ER-xL MEFs did not show statistically significant increase in passive ER Ca^2+^ leakage compared to WT control MEFs and Mt-xL MEFs (Fig. 4C, D). Moreover, as shown in Fig. 4E, F, Bcl-xL also appeared to have no significant impact on store-operated Ca^2+^ entry (SOCE), an important mechanism that promotes the entry of Ca^2+^ from the extracellular space to the ER in response to ER Ca^2+^ depletion [25, 26].

**Fig. 5:**
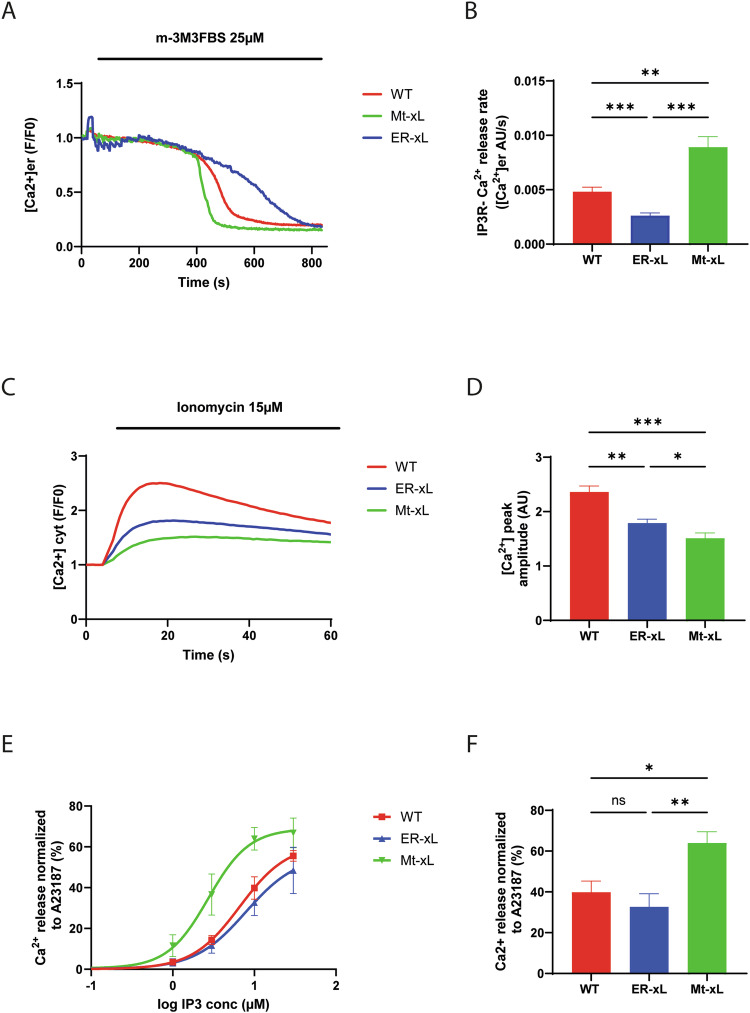
Bcl-xL at the ER interacts with IP3R and decreases IP3R Ca^2+^ permeability. **A** Representative curves of ER calcium release in WT, ER-xL and Mt-xL MEFs transfected with CEPIA-1er after 25μM m-3M3FBS (PLC agonist) injection. **B** Quantification of the slope coefficient of ER calcium release after PLC activation in MEFs. A Kruskall Wallis ‘s test was performed with N_WT_ = 46, N_ER-xL_ = 51, N_Mt-xL_ = 43 (mean ± SEM; **, *p* < 0.01; ***, *p* < 0.001). **C** Representative curves of cytosolic Ca^2+^ increase assessed with 5 μM Fluoforte after Ionomycin injection in WT, ER-xL and Mt-xL MEFs. **D** Quantification of Ionomycin response. The ratio of fluorescence, indicating Ca^2+^ peak amplitude relative to WT, is shown. A Kruskall Wallis’s test was performed with N_WT_ = 26, N_ER-xL_ = 26, N_Mt-xL_ = 26 (mean ± SEM; *, *p* < 0.05; **, *p* < 0.01; ***, *p* < 0.001). E-F. Quantification of ER calcium release normalized to A23187 maximal ionophore-induced Ca^2+^ release response through ^45^Ca^2+^ flux analysis. Dose-response curves are shown in **E**. Quantification performed at 10μM IP3 is shown in **F**. A one way ANOVA test was performed with N_WT_ = 5, N_ER-xL_ = 5, N_Mt-xL_ = 5 (mean ± SEM; ns, non-statistically significant; *, *p* < 0.05; **, *p* < 0.01). IP3-induced Ca^2+^ release in Mt-Bcl-xL MEFs is increased, compared to wild-type MEFs and ER-Bcl-xL.

We then evaluated the capacity of ER-xL to control IP3-dependent ER Ca^2+^ release. To do so, we activated IP3R by enhancing the production of IP3 through the Phospholipase C (PLC) activator m-3M3FBS and measured the decrease of the fluorescent ER-targeted Ca^2+^ indicator R-CEPIA1er fluorescence. The rate of IP3R-dependent ER Ca^2+^ release in ER-xL MEFs was significantly reduced (0.0026 AU/s) compared to WT control MEFs (0.0048 AU/s), corresponding to a 46% decrease (Fig. 5A, B). This inhibition of Ca^2+^ release from the ER was associated with a smaller increase in cytosolic Ca^2+^ levels after treatment with the Ca^2+^ ionophore ionomycin in ER-xL (1.79 AU) compared to WT control MEFs (2.36 AU, Fig. 5C, D). Notably, Mt-xL MEFs exhibited higher rates of IP3R-dependent ER Ca^2+^ release (0.0089 AU/s, Fig. 5A, B) together with a limited increase in cytosolic Ca^2+^ after ionomycin treatment (1.51 AU) compared to control MEFs (2.36 AU, Fig. 5C, D) which is consistent with previous studies reporting that mitochondrial Bcl-xL can promote VDAC-dependent Ca^2+^ uptake, which could limit the height of cytosolic calcium peaks [23]. In this respect, it should be noted that isolated mitochondria from Mito-xL MEFs show a high rate of Ca^2+^ uptake, compared to WT MEFS and ER-xL MEFs (Supplementary Fig. 4).

To directly assess the impact of Bcl-xL on IP3R-mediated ER Ca^2+^ release, we performed ^45^Ca^2+^ flux assays on plasma membrane-permeabilized MEF cells expressing WT Bcl-xL, ER-Bcl-xL or Mt-Bcl-XL. In these experiments, ER Ca^2+^ stores are loaded to steady state with ^45^Ca^2+^, while mitochondrial ^45^Ca^2+^ uptake is prevented by using NaN_3_. After reaching steady state ^45^Ca^2+^ loading, unidirectional passive Ca^2+^ leak from the ER is initiated by Thapsigargin. This is quantified as fractional loss (%/2 min) as a function of time. Once a constant fractional loss is obtained, cells are challenged with varying concentrations of IP3. The total releasable Ca^2+^, which was set to 100%, is determined by using A23187, a Ca^2+^ ionophore. IP3-induced ER Ca^2+^ release, normalized to A23187, was strongly sensitized for cells expressing Mt-Bcl-xL compared to WT-Bcl-xL, while it was similar for ER-Bcl-xL compared to WT-Bcl-xL (Fig. 5E, F & Supplementary Fig. 5). These data indicate that Bcl-XL present at ER membranes, in WT Bcl-xL as well as in ER-Bcl-xL-expressing cells, inhibits IP3Rs in comparison to Bcl-xL present at mitochondrial membranes. In this experimental analysis, IP3-induced Ca^2+^ release was similar for WT Bcl-xL versus ER-Bcl-xL, which may suggest that the majority of WT Bcl-xL is already present at ER membranes and inhibits IP3Rs or that the ER-resident fraction of Bcl-XL in the WT cells are sufficient to evoke IP3R inhibition. Moreover, this experiment is based on permeabilized cells, assessing unidirectional ER Ca^2+^ release in the absence of mitochondrial Ca^2+^ handling.

## Discussion

In addition to their role in regulating the mitochondrial pathway of apoptosis, non-canonical functions of Bcl-2 proteins emerged throughout the years [13]. Some of these functions are in part ensured through intracellular Ca^2+^ fluxes regulation [27, 28]. Indeed, although mitochondria are considered the major hub of Bcl-2 proteins, a number of Bcl-2 homologs, including Bcl-2 itself, can also localise to the ER where they exert opposing effects on ER-mediated Ca^2+^ handling [29]. Regarding Bcl-xL, although mainly involved in the mitochondrial pathway of apoptosis [30], a number of reports support that it contributes to a range of other processes, independent of apoptosis, including autophagy, migration, metabolism and signal transduction [29, 31]. Some of these non-canonical roles of Bcl-xL are presumably performed at the level of the ER [17, 32] although such a subcellular localisation has long been controversial. Indeed, Bcl-xL TM domain was reported to direct Bcl-xL towards the mitochondria, but not at the ER [30], whereas in contrast, Bcl-xL was shown to interact with the ER-based IP3R1 Ca^2+^ channel [16].

As an anti-apoptotic protein, Bcl-xL is a promising target regarding cancer therapy [8, 9, 11]. In this respect, understanding the molecular mechanisms that drive its subcellular localisation may help prevent unwanted side effects of Bcl-xL inhibitors, including BH3 mimetics [33, 34]. Here, we specifically interrogated mechanisms beyond the localisation of Bcl-xL at the ER and their involvement in ER Ca^2+^ turnover. Our observations about *bclx* KO MEFs’ vulnerability to cytotoxic drugs are in accordance with previous data about MOMP prevention by Bcl-xL [2]. Furthermore, we observed that *bclx* KO enhanced the UPR, following Ca^2+^-dependent insults, suggesting that Bcl-xL might be also involved in the control of Ca^2+^-dependent ER stress.

By generating knock-in mice expressing either ER- or mitochondria-targeted Bcl-xL, we aimed at further understanding the respective roles of both Bcl-xL subcellular pools. Although these models may have some limitations, in part because they presumably suppress the traffic of Bcl-xL between the different subcellular compartments [35], they have proved useful in addressing this issue.

We present some evidence that Staurosporine enhances cell death in ER-xL MEFs, compared to Mt-xL MEFs, indirectly confirming the cell death protection potential of mitochondrial Bcl-xL [19]. On the other hand, ER-xL MEFs were found to be more resistant to the ER stress-inducing agents Thapsigargin and Tunicamycin, compared to Mt-xL MEFs. Taken together our data indicate that ER-resident Bcl-xL prevents Ca^2+^-dependent stress by decreasing the release of Ca^2+^ from the ER lumen through IP3Rs. This is fully in line with previous observations showing that mitochondrial Bcl-xL is able to protect cells from ER stress by sequestering Bim at the mitochondria, subsequently reducing CHOP-induced apoptosis during plasma cell differentiation [36] and abrogating caspase 12-mediated apoptosis in murine myoblast cells [37]. Thus, ER-Bcl-xL and Mt-Bcl-xL may work synergistically to prevent ER-dependent cell death.

At the molecular level, Bcl-xL was reported to interact with the C-terminal domain of IP3R1 in the neighbourhood of its channel pore. Of note, Bcl-xL/IP3R interaction seems to be independent of the BH4 domain of Bcl-xL [18]. Through this interaction, Bcl-xL sensitizes IP3Rs to basal IP3, thereby promoting ER-to-mitochondria Ca^2+^ signaling [16, 17]. At higher Bcl-xL concentrations, Bcl-xL could also bind to the coupling domain of IP3Rs, thereby lowering ER Ca^2+^ release through the channel [16]. We confirmed these observations in MEFs using PLA. Furthermore, the Ca^2+^ flux measurements performed here suggest that ER-xL has limited effect on passive ER Ca^2+^release under non-stressful conditions. In contrast, Ca^2+^ fluxes measurement performed in the presence of high IP3 levels supported the notion that ER-xL prevents the full opening of IP3R Ca^2+^ channels in stressful conditions. This phenomenon might act as a survival defense mechanism in case of ER stress by limiting ER Ca^2+^ stores depletion and downstream UPR initiation. Actually, based on the duration and severity of the stress, unfolded proteins accumulate in ER lumen with concomitant decrease in ER Ca^2+^ levels. As a consequence, ER-stress sensors are activated, setting in motion adaptive responses that prompt either cell survival or cell death, depending on the severity and the duration of the stress [38].

**Fig. 6:**
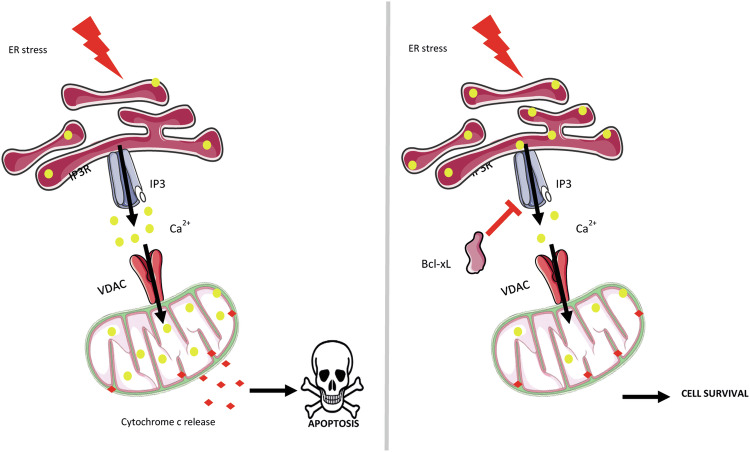
Bcl-xL reduces Ca^2+^-dependent ER stress by closing the IP3R channel. Proposed model for the role of Bcl-xL at the ER. Left panel: ER stress might induce ER Ca^2+^ release in the cytosol (yellow dots), promoting Ca^2+^ uptake by the mitochondria and subsequent apoptosis initiation through mPTP opening and cytochrome C release in the cytosol (red diamonds). Right panel: ER-targeted Bcl-xL interacts with IP3R and closes the channel, abrogating ER Ca^2+^ depletion and downstream apoptosis. This model highlights a new indirect anti-apoptotic function of Bcl-xL upon ER stress.

Together, our data reveal a new possible mode of action for the Bcl-xL at the ER (Fig. 6). According to the proposed model, upon induction of ER stress, Bcl-xL would negatively regulate IP3-mediated Ca^2+^ release and prevent ER Ca^2+^ depletion by preventing the opening of the IP3R channels. In this way, the ER pool of Bcl-xL would avert UPR initiation and downstream apoptosis, further promoting cell survival.

## Materials and methods

### Cell lines and drugs

Mouse Embryonic Fibroblasts (WT, ER-xL, and Mt-xL) were extracted from mice embryos at Embryonic day 13 (E13) and cultured along with *bclx KO* MEFs (kindly provided from Li laboratory, see [17]) under standard cell culture conditions (37˚C, 5% CO_2_) in Dulbecco’s modified Eagle’s medium (DMEM) high glucose medium (Gibco) supplemented with 10% Fetal Bovine Serum (FBS, Sigma Aldrich), 100 U/mL penicillin and 100 μg/mL streptomycin (Gibco). Several cytotoxic drugs, Ca^2+^ ionophores, channel inhibitors were used: Staurosporine (Sigma Aldrich), Etoposide (Sigma-Aldrich), Thapsigargin (Enzo Life Sciences), Tunicamycin (Sigma Aldrich), Ionomycin (Enzo Life Sciences), 2-APB (Tocris), Xestospongin C (Tocris), m-3M3FBS (Sigma Aldrich).

### Cell death detection

For cell death detection experiments, MEFs were analyzed using Incucyte ZOOM live-cell imaging system (Essen Bioscience). In brief, cell death was assessed using SYTOX Green^TM^ nucleic acid stain at 250 nM concentration (ThermoFisher Scientific) plus the addition of several cell death inducers: Staurosporine 1 μM, Etoposide 10 μM, Thapsigargin 1 μM, Tunicamycin 2 μg/mL. Images were automatically acquired in phase and green fluorescent channels every one hour for a maximum of 72 h at 4X magnification. Data were processed using a dedicated algorithm (Essen Bioscience). Cell proliferation was estimated using the phase-contrast images of the controls of the same experiments.

### Immunofluorescence

MEFs were seeded on a glass coverslip in 12-well plates at around 40% of confluence and transfected with ER-targeted EGFP. After cell attachment, the medium was discarded and the cells were incubated with mitochondria-staining dye (MitoTracker Red CMXRos, Life technologies) for 20 min at 37˚C then fixed with 4% Paraformaldehyde with 0.3% Triton X100. MEFs were washed 3 times with 0.1% Triton-PBS then incubated for 20 minutes (min) with blocking buffer (0.1% Triton X100, 3% BSA in PBS). Cells were subsequently incubated for 1 h with primary antibodies and 1 h with Alexa fluor 563 coupled secondary antibodies. Cells were washed 3 times after the incubation with both antibodies. Coverslips were mounted with a Dako mounting medium. Bcl-xL antibody (Cell Signaling #2764; 1/200) was used to detect endogenous Bcl-xL. For apoptosis assay after 250 nM Staurosporine treatment for 6 h or 4 µM Thapsigargin treatment for 24 h, the same procedure was performed without transfecting the MEFs. Cleaved Caspase 3 (Cell Signaling #9661, 1/1000) antibody was used to assess cell death followed by Alexa fluor 563 coupled secondary antibodies. Nuclei were detected with Hoechst 33342 dye (Invitrogen #H3570) at 10 μg/mL concentration. Images were acquired using a Zeiss 780 confocal microscope.

### Antibodies used for Western Blot

The following antibodies were used: Vinculin (Santa Cruz #sc-55465; 1/2000), Purified Mouse Anti-ATP Synthase β – F0F1 (BD Transduction Laboratories #612518; 1/2000), Calnexin (Cell signaling #2679; 1/1000), ATF4 (Cell Signaling #11815, 1/1000), CHOP (Cell Signaling #5554, 1/1000), Bcl-xL (Cell Signaling #2764, 1/1000). HRP-conjugated goat anti-mouse and goat anti-rabbit antibodies (DAKO) were used as secondary antibodies. Immunoblotting was performed according to standard procedures. For kinetic immunoblotting, MEFs were treated with the indicated drug and were harvested at each indicated time point where they were lysed and their protein content was measured.

### Subcellular fractionation

MEFs were harvested by trypsinisation and homogenized in MB buffer (210 mM mannitol, 70 mM sucrose, 1 mM EDTA and 10 mM HEPES (pH 7.4) containing protease inhibitors) by shearing (around 20 times) with a 1 mL syringe and a 26 G 5/8 needle. Homogenization was visually confirmed under the microscope. All steps were carried out at 4 °C. Cell extracts were centrifuged 3 times (10 min at 1500 g) to remove the nuclear fraction (pellet). Supernatants were then centrifuged 3 times (10 min at 10,600 g). After the third round of centrifugation, crude mitochondria pellet was carefully collected with a micropipette, suspended in MB buffer and a Percoll medium (225 mM mannitol, 25mM HEPES (pH 7.4), 1mM EGTA and 30% Percol -vol/vol-) and centrifuged for 30 min at 95000 g separating the MAMs and the mitochondria. The supernatant was centrifuged for 1 h at 100,000 g. After this last round of centrifugation, the obtained microsome pellet was resuspended in RIPA buffer. The purity of obtained fractions was assessed by Western blot.

### Intracellular Ca^2+^ measurements

For ER Ca^2+^ measurements, MEFs were transfected with 2 µg RCEPIA 1er probe 48 h before starting the experiment. Cells were seeded at 40% confluence in 8 well Nunc™ Lab-Tek™ II Chamber Slide and incubated with EM buffer (121 mM NaCl; 5.4 mM KCl, 2.6 mM MgCl2 hexahydrate, 6 mM NaHCO3, 5 mM D-Glucose, 25 mM HEPES pH 7.3). 10 µM Thapsigargin injections were done while quantifying the fluorescence with a Zeiss LSM 780 confocal microscope.

SOCE measurements were performed on MEFs incubated for 1 h with 5 µM Fluoforte dye, and washed with BBS containing 0.1 mM EGTA. After 20 seconds (s) of baseline fluorescence reading, Thapsigargin 10 µM was injected followed by 2 mM CaCl_2_ once the fluorescence drops back to baseline

For cytosolic Ca^2+^ measurements, MEFs were seeded onto a 96-well plate one day before the experiment. Cells were then loaded with 5 µM Fluoforte dye (Enzo Life Sciences) in EM buffer for 45 min at 37 °C followed by 2 washes with EM buffer. Fluorescence was assessed using the ClarioSTAR plate reader, following 15 µM Ionomycin injections (Enzo Life Sciences).

For IP3R-induced Ca^2+^ release, transfected MEFs with RCEPIA 1er probe were injected with 25 µM m-3M3FBS (Sigma-Aldrich), direct activator of PLC. The Fluorescence was assessed using Zeiss LSM 780 confocal microscope. The speed of Ca^2+^ release was calculated by measuring the slope of the increase of fluorescence upon injection.

### Mitochondrial Ca^2+^ uptake

Mitochondrial Ca^2+^ uptake on isolated mitochondria was performed using Oregon Green 5 N hexapotassium salt. For each condition (WT, ER-xL, Mt-xL), two 10 cm petri dishes were washed twice with PBS and cells were scraped with 1 mL ice cold MB buffer (210 mM mannitol, 70 mM sucrose, 1 mM EDTA, 10 mM HEPES [pH 7.5], containing proteases inhibitors). The cells were disrupted with a 1 mL syringue and a 26 G x 2/3 needle 50 times on ice. The cell lysate was centrifuged twice at 1500 g for 10 min at 4 °C to eliminate nuclei. The supernatant was spun twice at 10,600 g for 10 min at 4 °C to pellet mitochondria. Purified mitochondria were resuspended in KCl medium (125 mM KCl, 2 mM K2HPO4, 1 mM MgCl2 and 20 mM HEPES, pH 7) containing 1 μM Oregon Green 5 N (Molecular Probes) and supplemented with 5 mM glutamate and 5 mM malate. Ca^2+^ measurements were performed using a Clariostar microplate fluorescence reader (Ex/Em = 492/517 nm). Ca^2+^ was injected 30 s after the begining of the measurement at a final concentration of 20 μM.

### ImmunoGold labelling

Subcellular localisation of endogenous Bcl-xL in WT, ER-xL, Mt-xL and *bclx* KO MEFs was performed by Bcl-xL ImmunoGold labeling and Transmission Electron Microscopy (TEM) imaging. Uranyle acetate staining was used to allow for subcellular structure visualization in fixed cell sections. Images were collected using a JEOL 1400JEM transmission electron microscope and Zeiss LSM780 confocal microscope.

### Proximity ligation assay (PLA)

MEFs were grown on coverslips and fixed with methanol for 2 min. After two PBS washes, MEFs were saturated with the blocking solution followed by a 1 h at 37 °C incubation with the primary antibodies: Bcl-xL (Cell Signaling #2764, 1/1000) and IP3R C-terminal (abcam #ab190239, 1/1000). PLA secondary antibodies probes were added and incubated for 1 h at 37 °C after 3 PBS washes. Ligation and amplification (100 min at 37 °C) steps followed. Cells were mounted with Duolink II Mounting medium containing DAPI and images were acquired using the fluorescence microscope.

### ^45^Ca^2+^ assays

^45^Ca^2+^ flux analysis was performed as previously described [39, 40]. After permeabilizing the MEFs, IP3R-mediated Ca^2+^ release was assessed through the addition of increasing concentrations of IP3. Non-mitochondrial stores were loaded to steady state with ^45^Ca^2+^. After washing the cells with efflux medium containing thapsigargin (4 mM), unidirectional ER ^45^Ca^2+^ efflux was monitored for 18 min and the replacing the efflux medium every 2 min. After reaching steady-state ^45^Ca^2+^efflux, varying concentrations of IP3 was added At the end of the experiments, 2% sodium dodecyl sulfate (SDS) was added for 30 min to obtain the ^45^Ca^2+^ remaining in the stores. Ca^2+^ release is plotted as fractional loss (%/2 min), obtained by measuring the amount of Ca^2+^ released in 2 min divided by the total store Ca^2+^ content at that time. The IP3-sensitive Ca^2+^ release was quantified as the difference in fractional loss after and before the addition of IP3. Graphpad prism 10.2.0 was used to analyze, plot, and fit the data points. Dose–response curves using different concentrations of IP3 were obtained by normalizing all data points to the A23187 ionophore-induced Ca^2+^-release values, which was set at 100%.

### Cell cycle FACS analysis

FACS analysis with Propidium Iodide (PI) (Abcam) was carried out by flow cytometry (BD FACSCanto™ II, BD Bioscience) according to manufacturer’s recommended protocol. Briefly, MEFs were seeded and grown overnight onto a 12-well plate. Afterwards, they were trypsinized and harvested. The obtained cell pellet was washed with PBS and fixed with cold ethanol for 30 min at 4 °C. After 2 PBS washes, MEFs were treated with 50 µl of a 100 µg/ml stock of RNase to ensure that only DNA is stained. 200 µL of PI (from 50 µg/ml stock solution) was then added and the cell cycle was analyzed by flow cytometry (BD FACSCanto™ II, BD Bioscience) using untreated cells as negative control for gating. A total of 10.000 events were recorded during the experiments (*n* = 3). The percentage of each cell population was analyzed using BD FACSDIVA 8.0.1 software (BD Bioscience, USA).

### Genotyping by PCR

PCR-based analysis was used to genotype mice. Each reaction mixture consisted of deoxynucleoside triphosphates, MgCl_2_, a forward primer specific for Bcl-xL exon 3: EXE-F (5’-GGAAAGGCCAGGAGCGCCTTC-3’), a reverse complementary primer specific for Bcl-xL exon 3: XTAG-R (5’-CCCAACCCTGTGATAGGGCAAG-3’), Taq polymerase, specific buffer and genomic DNA. Conditions for PCR were as follow: (1) 5 min at 98 °C; (2) 35 cycles, with 1 cycle consisting of 30 s at 94 °C, 30 s at 59 °C, and 40 s at 72 °C and (3) an elongation period of 5 min at 72 °C. Amplified products were analyzed by electrophoresis in 2% agarose gels and bands were visualized by Gel-Doc BIO-RAD imaging system.

### Statistical analyses

Histograms show average values ± SEM or average values ± SD. The normal distribution was calculated with Shapiro-Wilk’s test. All statistical analyses were two-sided. To compare the variances between the samples analysed, a Brown-Forsythe test, a Bartlett’s test or an F-test was performed, depending on the size of the samples. The statistical tests used and the number of replicates are indicated in the legends of the figures.

### Supplementary information

Supplementary figures

Raw data related to western blotting experiments

## References

[1] Boise LH, González-García M, Postema CE, Ding L, Lindsten T, Turka LA, et al. bcl-x, a bcl-2-related gene that functions as a dominant regulator of apoptotic cell death. Cell. 1993;74:597–608. 8358789 10.1016/0092-8674(93)90508-N

[2] Shamas-Din A, Kale J, Leber B, Andrews DW. Mechanisms of action of Bcl-2 family proteins. Cold Spring Harb. Perspect. Biol. 2013;5:a008714. 23545417 10.1101/cshperspect.a008714 PMC3683897

[3] Fulda S, Debatin K-M. Extrinsic versus intrinsic apoptosis pathways in anticancer chemotherapy. Oncogene. 2006;25:4798–811. 16892092 10.1038/sj.onc.1209608

[4] Vandenabeele P, Bultynck G, Savvides SN. Pore-forming proteins as drivers of membrane permeabilization in cell death pathways. Nat. Rev. Mol. Cell Biol. 2023;24:312–33. 36543934 10.1038/s41580-022-00564-w

[5] Watanabe J, Kushihata F, Honda K, Mominoki K, Matsuda S, Kobayashi N. Bcl-xL overexpression in human hepatocellular carcinoma. Int J. Oncol. 2002;21:515–9. 12168094

[6] Gobé G, Rubin M, Williams G, Sawczuk I, Buttyan R. Apoptosis and expression of Bcl-2, Bcl-XL, and Bax in renal cell carcinomas. Cancer Invest. 2002;20:324–32. 12025227 10.1081/CNV-120001177

[7] Ghaneh P, Kawesha A, Evans JD, Neoptolemos JP. Molecular prognostic markers in pancreatic cancer. J. Hepatob Pancreat. Surg. 2002;9:1–11. 10.1007/s005340200000 12021893

[8] Weiler M, Bähr O, Hohlweg U, Naumann U, Rieger J, Huang H, et al. BCL-xL: time-dependent dissociation between modulation of apoptosis and invasiveness in human malignant glioma cells. Cell Death Differ. 2006;13:1156–69. 16254573 10.1038/sj.cdd.4401786

[9] Koehler BC, Scherr A-L, Lorenz S, Urbanik T, Kautz N, Elssner C, et al. Beyond cell death - antiapoptotic Bcl-2 proteins regulate migration and invasion of colorectal cancer cells in vitro. PLoS ONE. 2013;8:e76446. 24098503 10.1371/journal.pone.0076446 PMC3789675

[10] Martin SS, Ridgeway AG, Pinkas J, Lu Y, Reginato MJ, Koh EY, et al. A cytoskeleton-based functional genetic screen identifies Bcl-xL as an enhancer of metastasis, but not primary tumor growth. Oncogene. 2004;23:4641–5. 15064711 10.1038/sj.onc.1207595

[11] Choi S, Chen Z, Tang LH, Fang Y, Shin SJ, Panarelli NC, et al. Bcl-xL promotes metastasis independent of its anti-apoptotic activity. Nat. Commun. 2016;7:1–13. 10.1038/ncomms10384 PMC4735924 26785948

[12] Popgeorgiev N, Jabbour L, Gillet G. Subcellular Localization and Dynamics of the Bcl-2 Family of Proteins. Front Cell Dev. Biol. 2018;6:13. 29497611 10.3389/fcell.2018.00013 PMC5819560

[13] Bonneau B, Prudent J, Popgeorgiev N, Gillet G. Biochimica et Biophysica Acta. BBA - Mol. Cell Res. 2013;1833:1755–65. 10.1016/j.bbamcr.2013.01.021 23360981

[14] Rong Y-P, Aromolaran AS, Bultynck G, Zhong F, Li X, McColl K, et al. Targeting Bcl-2-IP3 Receptor Interaction to Reverse Bcl-2’s Inhibition of Apoptotic Calcium Signals. Mol. Cell. 2008;31:255–65. 18657507 10.1016/j.molcel.2008.06.014 PMC3660092

[15] Ivanova H, Wagner LE, Tanimura A, Vandermarliere E, Luyten T, Welkenhuyzen K, et al. Bcl-2 and IP3 compete for the ligand-binding domain of IP3Rs modulating Ca2+ signaling output. Cell Mol. Life Sci. 2019;76:3843–59. 30989245 10.1007/s00018-019-03091-8 PMC11105292

[16] Yang J, Vais H, Gu W, Foskett JK. Biphasic regulation of InsP 3receptor gating by dual Ca 2+release channel BH3-like domains mediates Bcl-x Lcontrol of cell viability. Proc. Natl Acad. Sci. USA. 2016;113:E1953–62. 26976600 10.1073/pnas.1517935113 PMC4822637

[17] White C, Li C, Yang J, Petrenko NB, Madesh M, Thompson CB, et al. The endoplasmic reticulum gateway to apoptosis by Bcl-X(L) modulation of the InsP3R. Nat. Cell Biol. 2005;7:1021–8. 16179951 10.1038/ncb1302 PMC2893337

[18] Rosa N, Ivanova H, Wagner LE, Kale J, La Rovere R, Welkenhuyzen K, et al. Bcl-xL acts as an inhibitor of IP3R channels, thereby antagonizing Ca2+-driven apoptosis. Cell Death Differ. 2022;29:788–805. 34750538 10.1038/s41418-021-00894-w PMC8990011

[19] Eno CO, Eckenrode EF, Olberding KE, Zhao G, White C, Li C. Distinct roles of mitochondria- and ER-localized Bcl-xL in apoptosis resistance and Ca2+ homeostasis. Mol. Biol. Cell. 2012;23:2605–18. 22573883 10.1091/mbc.e12-02-0090 PMC3386223

[20] Motoyama. Massive Cell Death of Immature Hematopoietic Cells and Neurons in Bcl-x-Deficient Mice. Science (New York, NY) 1995:1–5. 10.1126/science.7878471 7878471

[21] Nguyen TTM, Gadet R, Lanfranchi M, Lahaye RA, Yandiev S, Lohez O, et al. Mitochondrial Bcl-xL promotes brain synaptogenesis by controlling non-lethal caspase activation. iScience. 2023;26:106674. 37182099 10.1016/j.isci.2023.106674 PMC10173740

[22] Hetz C. The unfolded protein response: controlling cell fate decisions under ER stress and beyond. Nat. Rev. Mol. Cell Biol. 2012;13:89–102. 22251901 10.1038/nrm3270

[23] Huang H, Hu X, Eno CO, Zhao G, Li C, White C. An interaction between Bcl-xL and the voltage-dependent anion channel (VDAC) promotes mitochondrial Ca2+ uptake. J. Biol. Chem. 2013;288:19870–81. 23720737 10.1074/jbc.M112.448290 PMC3707689

[24] Decuypere J-P, Kindt D, Luyten T, Welkenhuyzen K, Missiaen L, De Smedt H, et al. mTOR-Controlled Autophagy Requires Intracellular Ca(2+) Signaling. PLoS ONE. 2013;8:e61020. 23565295 10.1371/journal.pone.0061020 PMC3614970

[25] Putney JW. Capacitative calcium entry: sensing the calcium stores. J. Cell Biol. 2005;169:381–2. 15866892 10.1083/jcb.200503161 PMC2171919

[26] Clapham DE. Calcium signaling. Cell. 2007;131:1047–58. 18083096 10.1016/j.cell.2007.11.028

[27] De Smet P, Parys JB, Callewaert G, Weidema AF, Hill E, De Smedt H, et al. Xestospongin C is an equally potent inhibitor of the inositol 1,4,5-trisphosphate receptor and the endoplasmic-reticulum Ca(2+) pumps. Cell Calcium. 1999;26:9–13. 10892566 10.1054/ceca.1999.0047

[28] Vervliet T, Parys JB, Bultynck G. Bcl-2 proteins and calcium signaling: complexity beneath the surface. Oncogene. 2016;35:5079–92. 26973249 10.1038/onc.2016.31

[29] Hardwick JM, Soane L. Multiple Functions of BCL-2 Family Proteins. Cold Spring Harb. Perspect. Biol. 2013;5:a008722. 23378584 10.1101/cshperspect.a008722 PMC3552500

[30] Kaufmann T, Schlipf S, Sanz J, Neubert K, Stein R, Borner C. Characterization of the signal that directs Bcl-x(L), but not Bcl-2, to the mitochondrial outer membrane. J. Cell Biol. 2003;160:53–64. 12515824 10.1083/jcb.200210084 PMC2172731

[31] Carné Trécesson Sde, Souazé F, Basseville A, Bernard A-C, Pécot J, Lopez J, et al. BCL-XL directly modulates RAS signalling to favour cancer cell stemness. Nat. Commun. 2017;8:1123. 29066722 10.1038/s41467-017-01079-1 PMC5654832

[32] Vervliet T, Lemmens I, Vandermarliere E, Decrock E, Ivanova H, Monaco G, et al. Ryanodine receptors are targeted by anti-apoptotic Bcl-XL involving its BH4 domain and Lys87 from its BH3 domain. Nat. Publ. Group. 2015;5:9641. 10.1038/srep09641 PMC4397538 25872771

[33] Mason KD, Carpinelli MR, Fletcher JI, Collinge JE, Hilton AA, Ellis S, et al. Programmed anuclear cell death delimits platelet life span. Cell. 2007;128:1173–86. 17382885 10.1016/j.cell.2007.01.037

[34] Schoenwaelder SM, Jarman KE, Gardiner EE, Hua M, Qiao J, White MJ, et al. Bcl-xL-inhibitory BH3 mimetics can induce a transient thrombocytopathy that undermines the hemostatic function of platelets. Blood. 2011;118:1663–74. 21673344 10.1182/blood-2011-04-347849

[35] Edlich F, Banerjee S, Suzuki M, Cleland MM, Arnoult D, Wang C, et al. Bcl-x(L) retrotranslocates Bax from the mitochondria into the cytosol. Cell. 2011;145:104–16. 21458670 10.1016/j.cell.2011.02.034 PMC3070914

[36] Gaudette BT, Iwakoshi NN, Boise LH. Bcl-xL protein protects from C/EBP homologous protein (CHOP)-dependent apoptosis during plasma cell differentiation. J. Biol. Chem. 2014;289:23629–40. 25023286 10.1074/jbc.M114.569376 PMC4156059

[37] Morishima N, Nakanishi K, Tsuchiya K, Shibata T, Seiwa E. Translocation of Bim to the endoplasmic reticulum (ER) mediates ER stress signaling for activation of caspase-12 during ER stress-induced apoptosis. J. Biol. Chem. 2004;279:50375–81. 15452118 10.1074/jbc.M408493200

[38] Mekahli D, Bultynck G, Parys JB, De Smedt H, Missiaen L Endoplasmic-reticulum calcium depletion and disease. *Cold Spring* Harbor Perspect Biol. 2011;3. 10.1101/cshperspect.a004317. 10.1101/cshperspect.a004317 PMC3098671 21441595

[39] Missiaen L, De Smedt H, Droogmans G, Casteels R. Ca2+ release induced by inositol 1,4,5-trisphosphate is a steady-state phenomenon controlled by luminal Ca2+ in permeabilized cells. Nature. 1992;357:599–602. 1608471 10.1038/357599a0

[40] Missiaen L, De Smedt H, Parys JB, Raeymaekers L, Droogmans G, Van Den Bosch L, et al. Kinetics of the non-specific calcium leak from non-mitochondrial calcium stores in permeabilized A7r5 cells. Biochem J. 1996;317:849–53. 8760372 10.1042/bj3170849 PMC1217562

