# Supplementary material for: The endoplasmic reticulum pool of Bcl-xL prevents cell death through IP3R-dependent calcium release
Source: Cell Death Discov. 2024 Aug 1;10:346. doi: 10.1038/s41420-024-02112-1 (PMC11294475; doi:10.1038/s41420-024-02112-1)
Supplement: Supplementary file 1 — Supplementary figures [file 41420_2024_2112_MOESM1_ESM.pptx]

## Slide 1
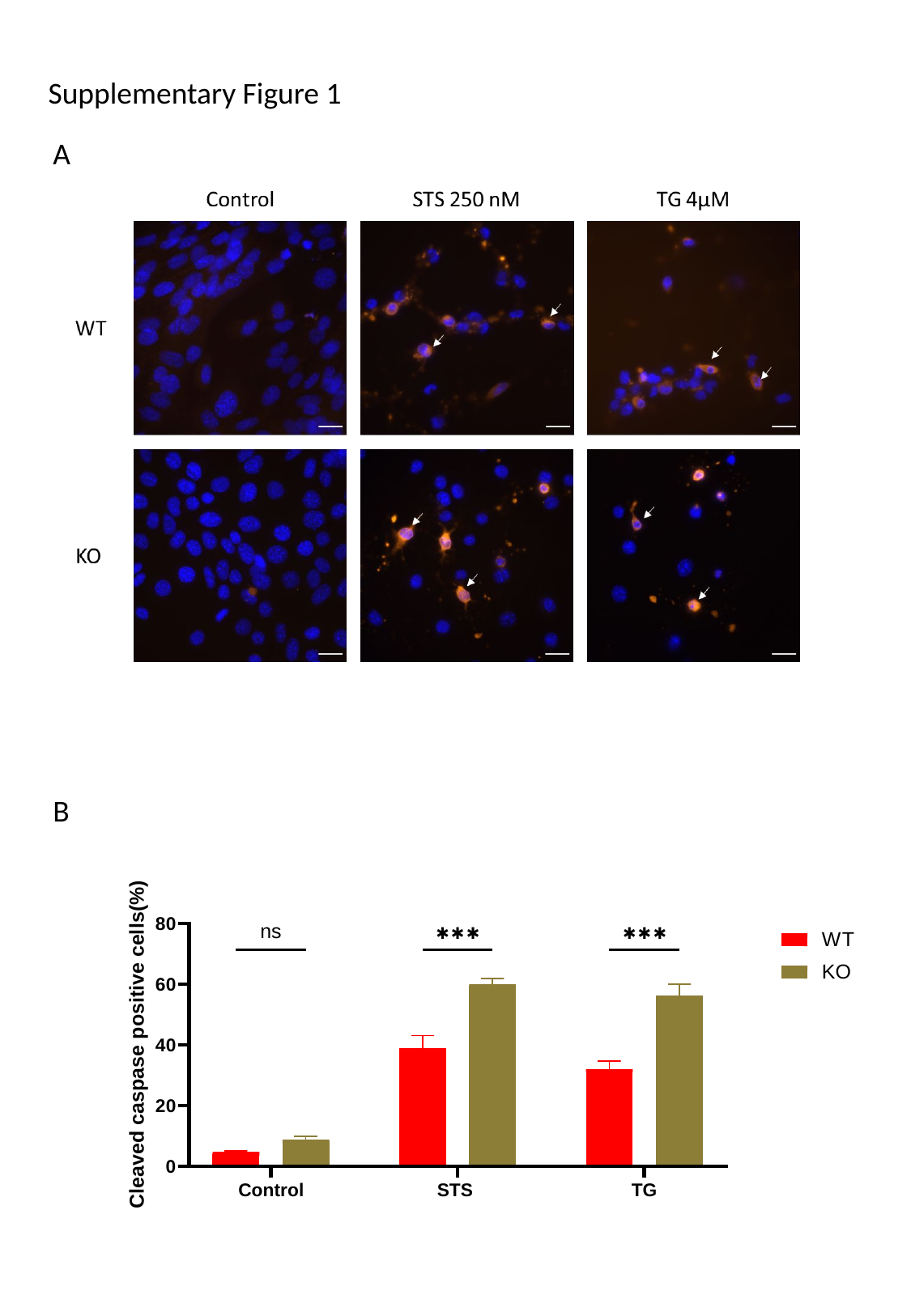

Supplementary Figure 1
A
B

## Slide 2
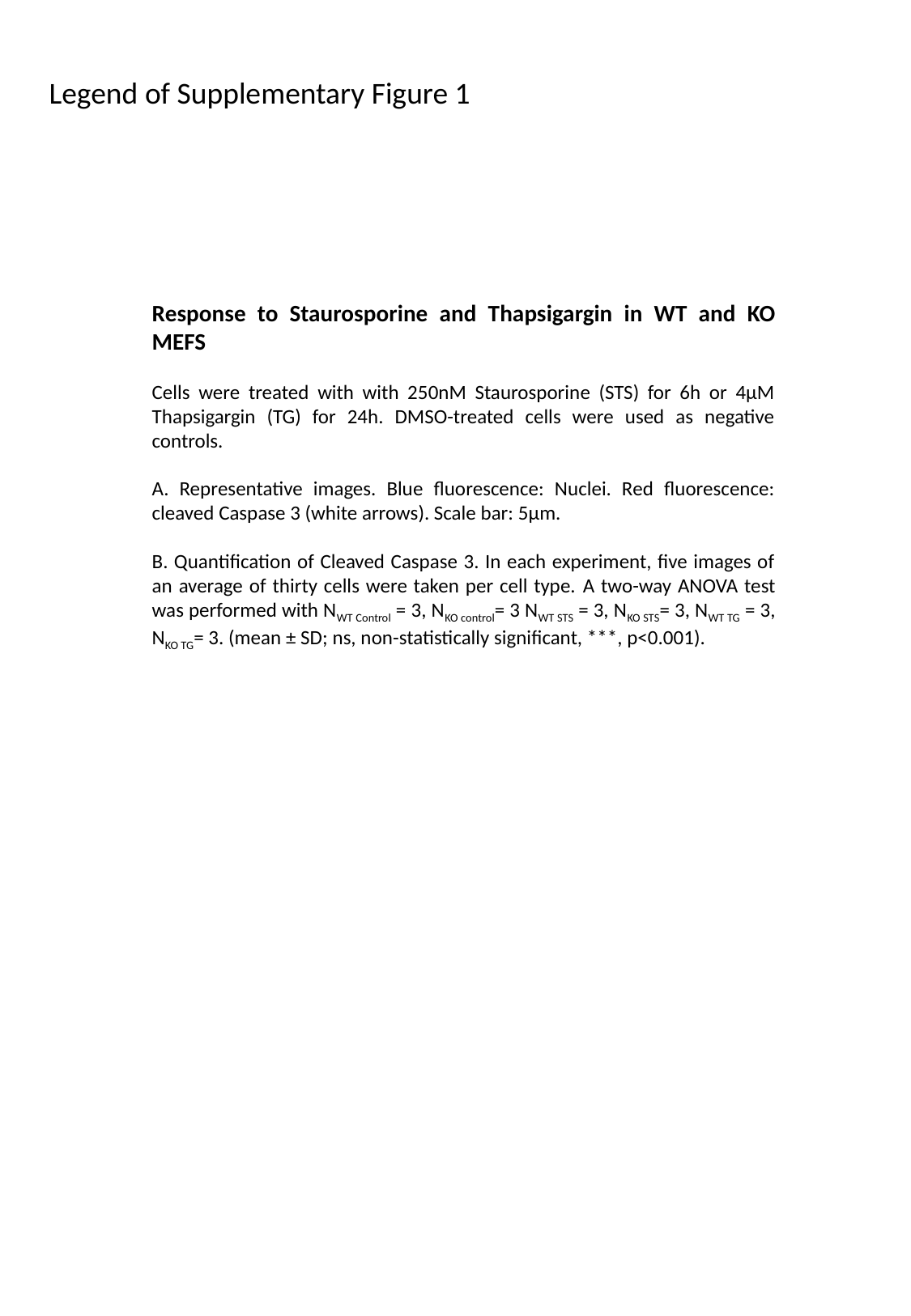

Legend of Supplementary Figure 1
Response to Staurosporine and Thapsigargin in WT and KO MEFS
Cells were treated with with 250nM Staurosporine (STS) for 6h or 4µM Thapsigargin (TG) for 24h. DMSO-treated cells were used as negative controls.
A. Representative images. Blue fluorescence: Nuclei. Red fluorescence: cleaved Caspase 3 (white arrows). Scale bar: 5µm.
B. Quantification of Cleaved Caspase 3. In each experiment, five images of an average of thirty cells were taken per cell type. A two-way ANOVA test was performed with NWT Control = 3, NKO control= 3 NWT STS = 3, NKO STS= 3, NWT TG = 3, NKO TG= 3. (mean ± SD; ns, non-statistically significant, ***, p<0.001).

## Slide 3
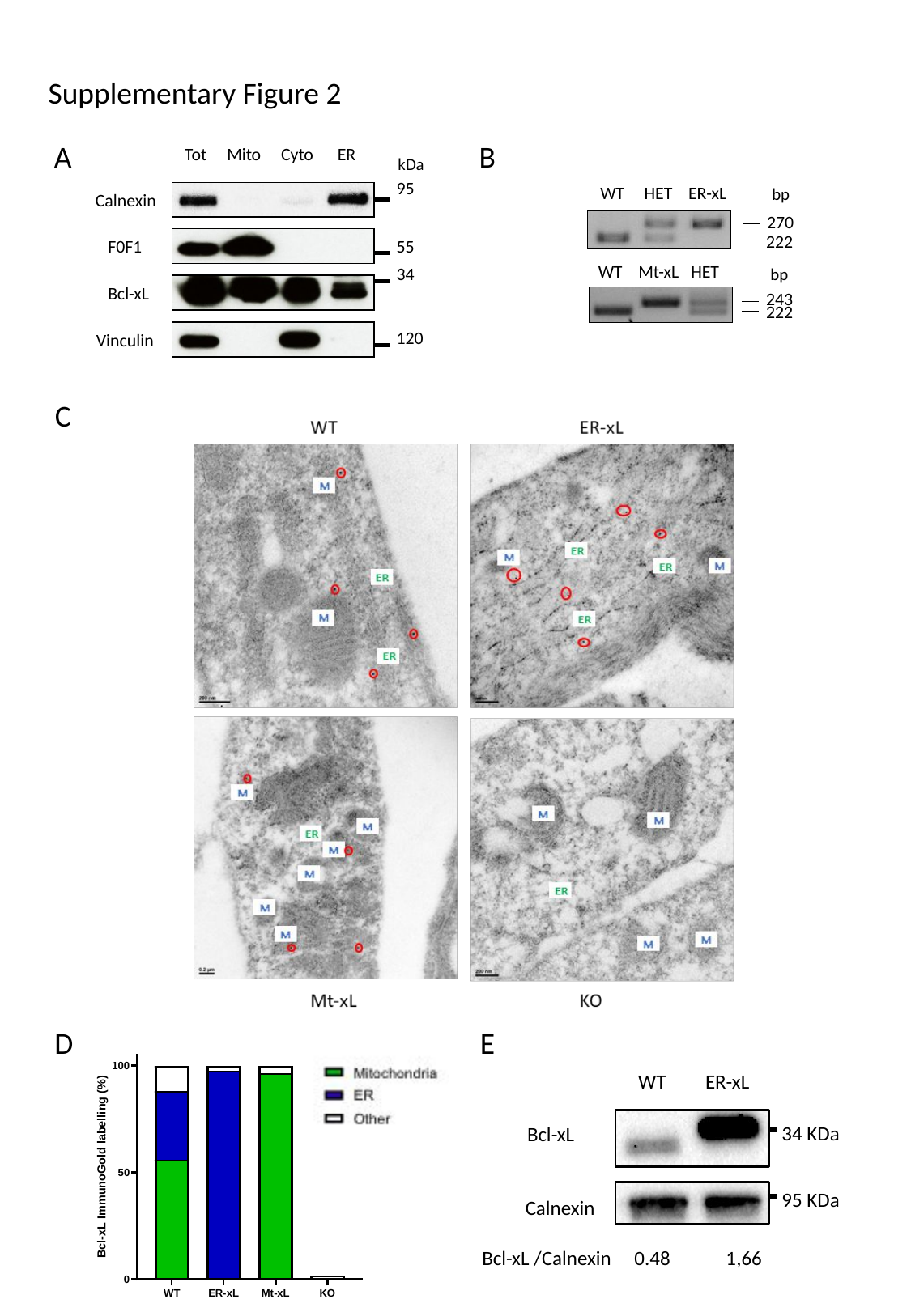

Supplementary Figure 2
A
B
Tot Mito Cyto ER
kDa
95
Calnexin
55
F0F1
34
Bcl-xL
120
Vinculin
 WT HET ER-xL
bp
270
222
 WT Mt-xL HET
bp
243
222
C
D
E
WT
ER-xL
34 KDa
Bcl-xL
95 KDa
Calnexin
Bcl-xL /Calnexin 0.48 1,66

## Slide 4
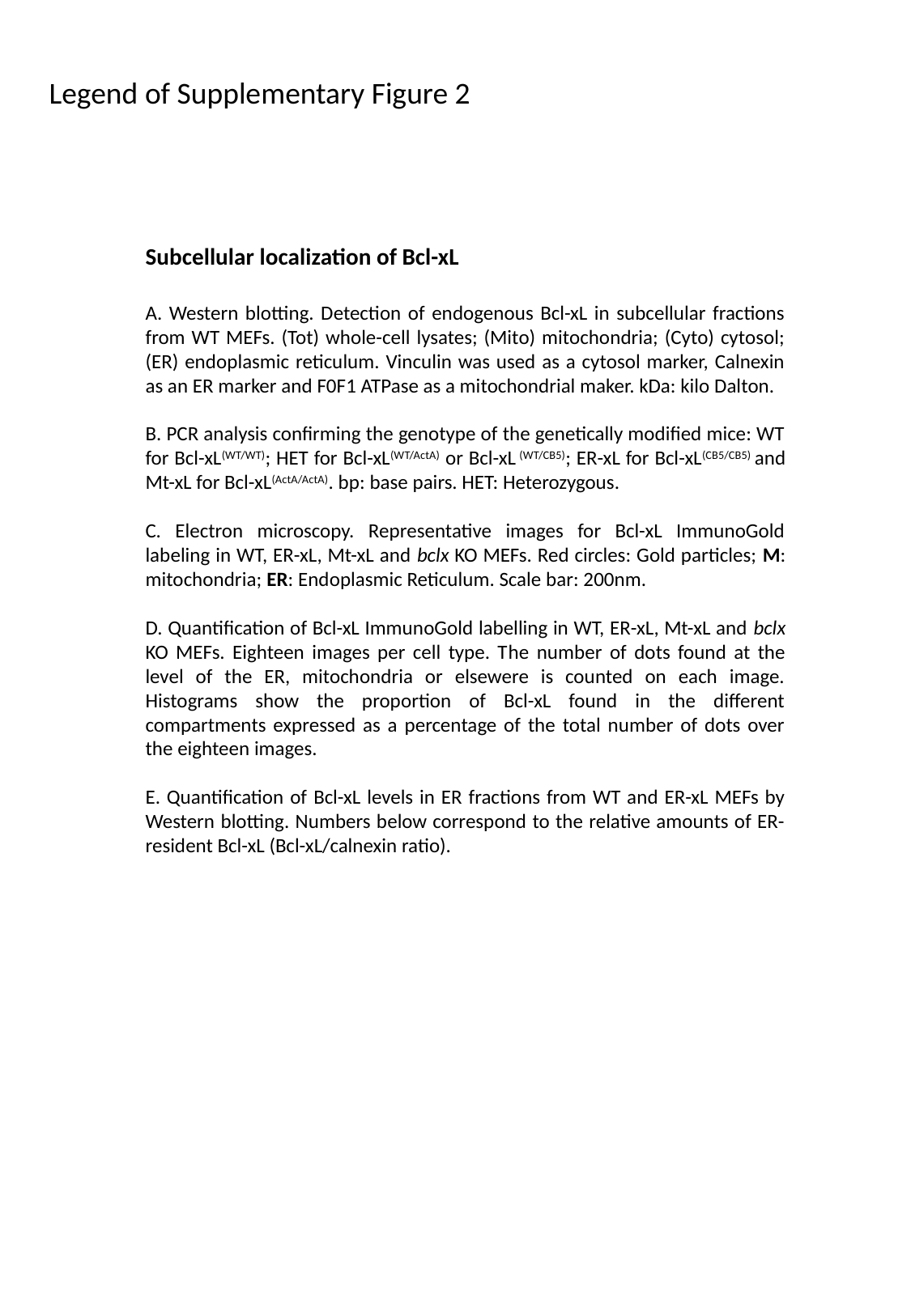

Legend of Supplementary Figure 2
Subcellular localization of Bcl-xL
A. Western blotting. Detection of endogenous Bcl-xL in subcellular fractions from WT MEFs. (Tot) whole-cell lysates; (Mito) mitochondria; (Cyto) cytosol; (ER) endoplasmic reticulum. Vinculin was used as a cytosol marker, Calnexin as an ER marker and F0F1 ATPase as a mitochondrial maker. kDa: kilo Dalton.
B. PCR analysis confirming the genotype of the genetically modified mice: WT for Bcl-xL(WT/WT); HET for Bcl-xL(WT/ActA) or Bcl-xL (WT/CB5); ER-xL for Bcl-xL(CB5/CB5) and Mt-xL for Bcl-xL(ActA/ActA). bp: base pairs. HET: Heterozygous.
C. Electron microscopy. Representative images for Bcl-xL ImmunoGold labeling in WT, ER-xL, Mt-xL and bclx KO MEFs. Red circles: Gold particles; M: mitochondria; ER: Endoplasmic Reticulum. Scale bar: 200nm.
D. Quantification of Bcl-xL ImmunoGold labelling in WT, ER-xL, Mt-xL and bclx KO MEFs. Eighteen images per cell type. The number of dots found at the level of the ER, mitochondria or elsewere is counted on each image. Histograms show the proportion of Bcl-xL found in the different compartments expressed as a percentage of the total number of dots over the eighteen images.
E. Quantification of Bcl-xL levels in ER fractions from WT and ER-xL MEFs by Western blotting. Numbers below correspond to the relative amounts of ER-resident Bcl-xL (Bcl-xL/calnexin ratio).

## Slide 5
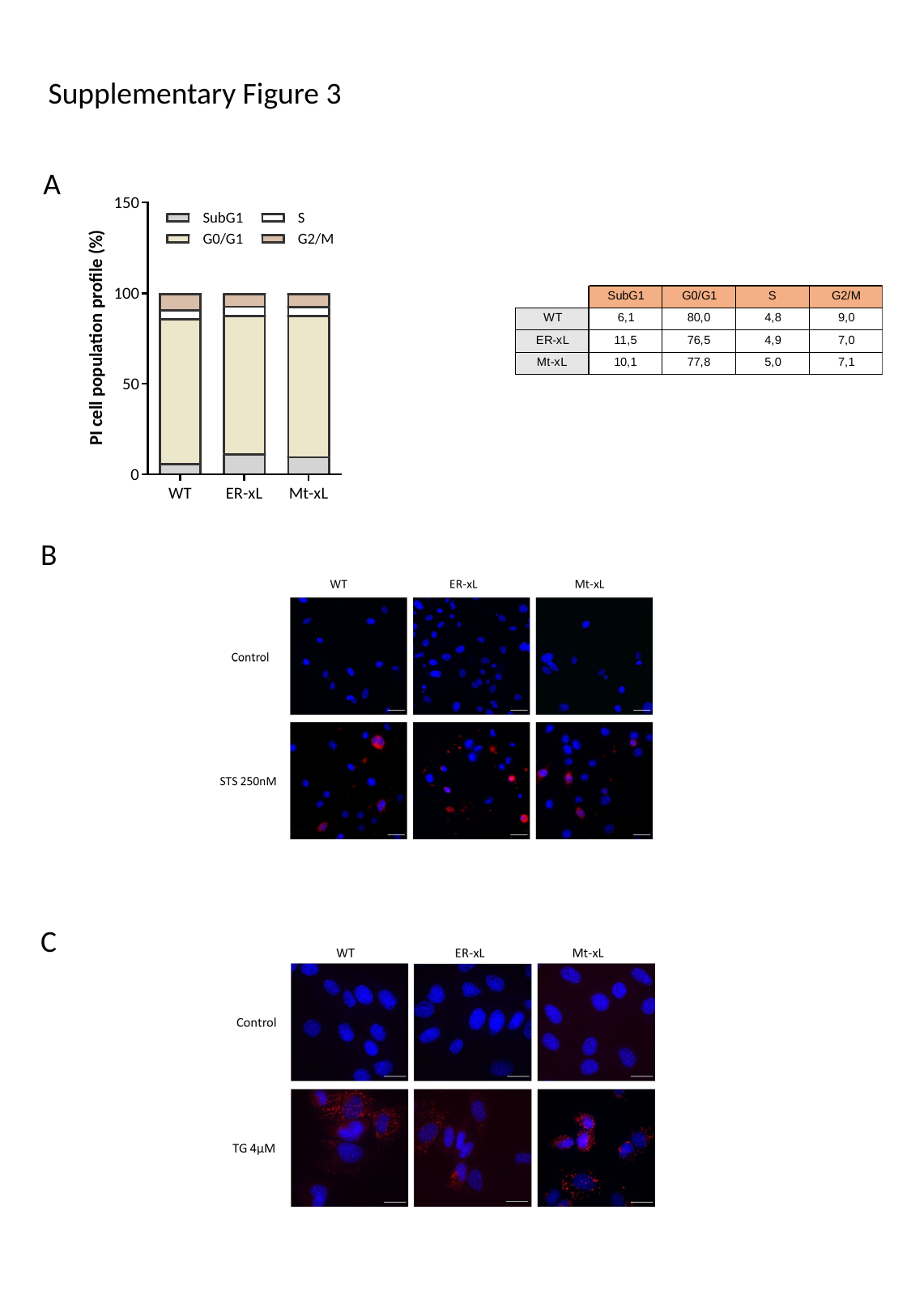

Supplementary Figure 3
A
B
C

## Slide 6
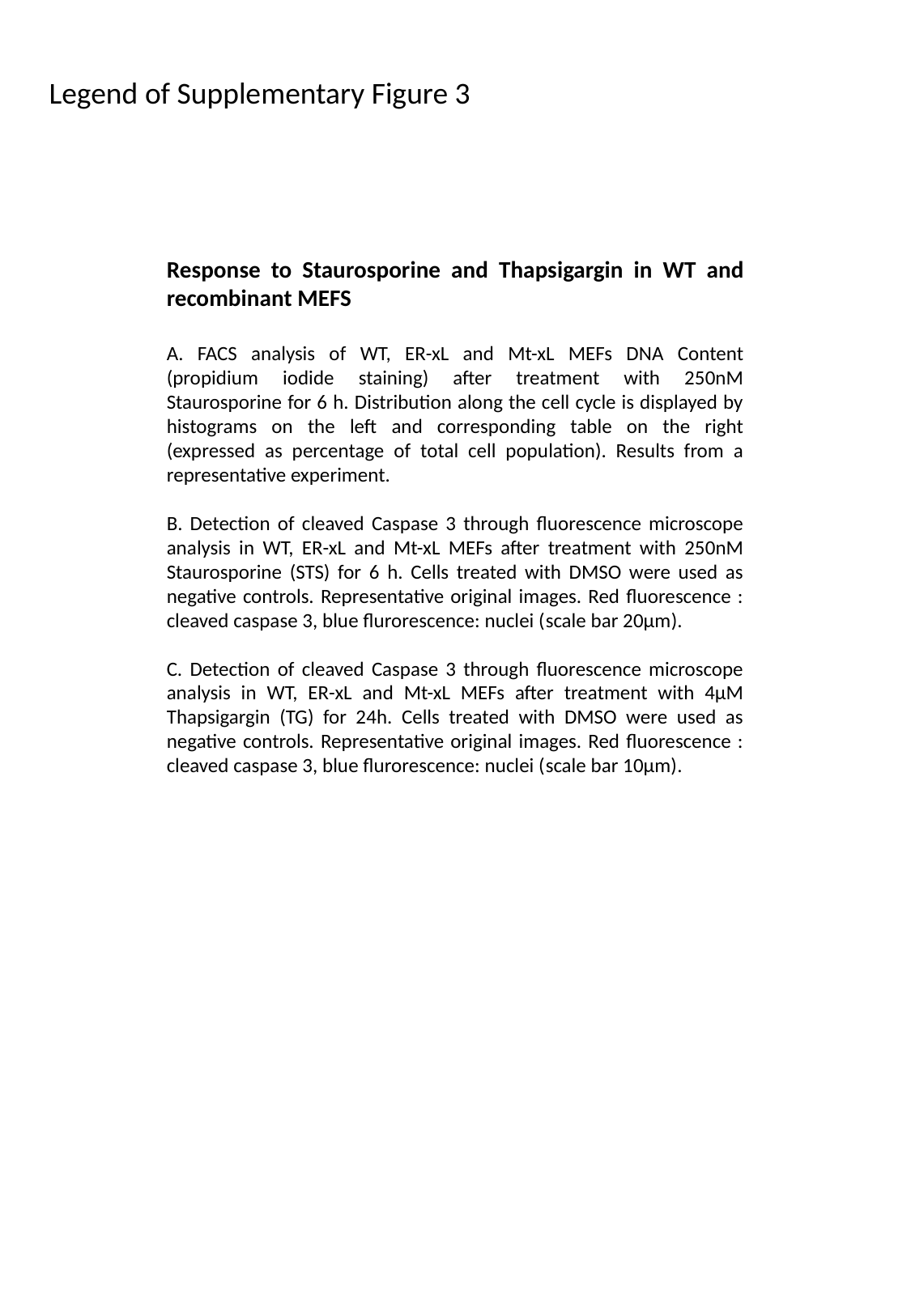

Legend of Supplementary Figure 3
Response to Staurosporine and Thapsigargin in WT and recombinant MEFS
A. FACS analysis of WT, ER-xL and Mt-xL MEFs DNA Content (propidium iodide staining) after treatment with 250nM Staurosporine for 6 h. Distribution along the cell cycle is displayed by histograms on the left and corresponding table on the right (expressed as percentage of total cell population). Results from a representative experiment.
B. Detection of cleaved Caspase 3 through fluorescence microscope analysis in WT, ER-xL and Mt-xL MEFs after treatment with 250nM Staurosporine (STS) for 6 h. Cells treated with DMSO were used as negative controls. Representative original images. Red fluorescence : cleaved caspase 3, blue flurorescence: nuclei (scale bar 20µm).
C. Detection of cleaved Caspase 3 through fluorescence microscope analysis in WT, ER-xL and Mt-xL MEFs after treatment with 4µM Thapsigargin (TG) for 24h. Cells treated with DMSO were used as negative controls. Representative original images. Red fluorescence : cleaved caspase 3, blue flurorescence: nuclei (scale bar 10µm).

## Slide 7
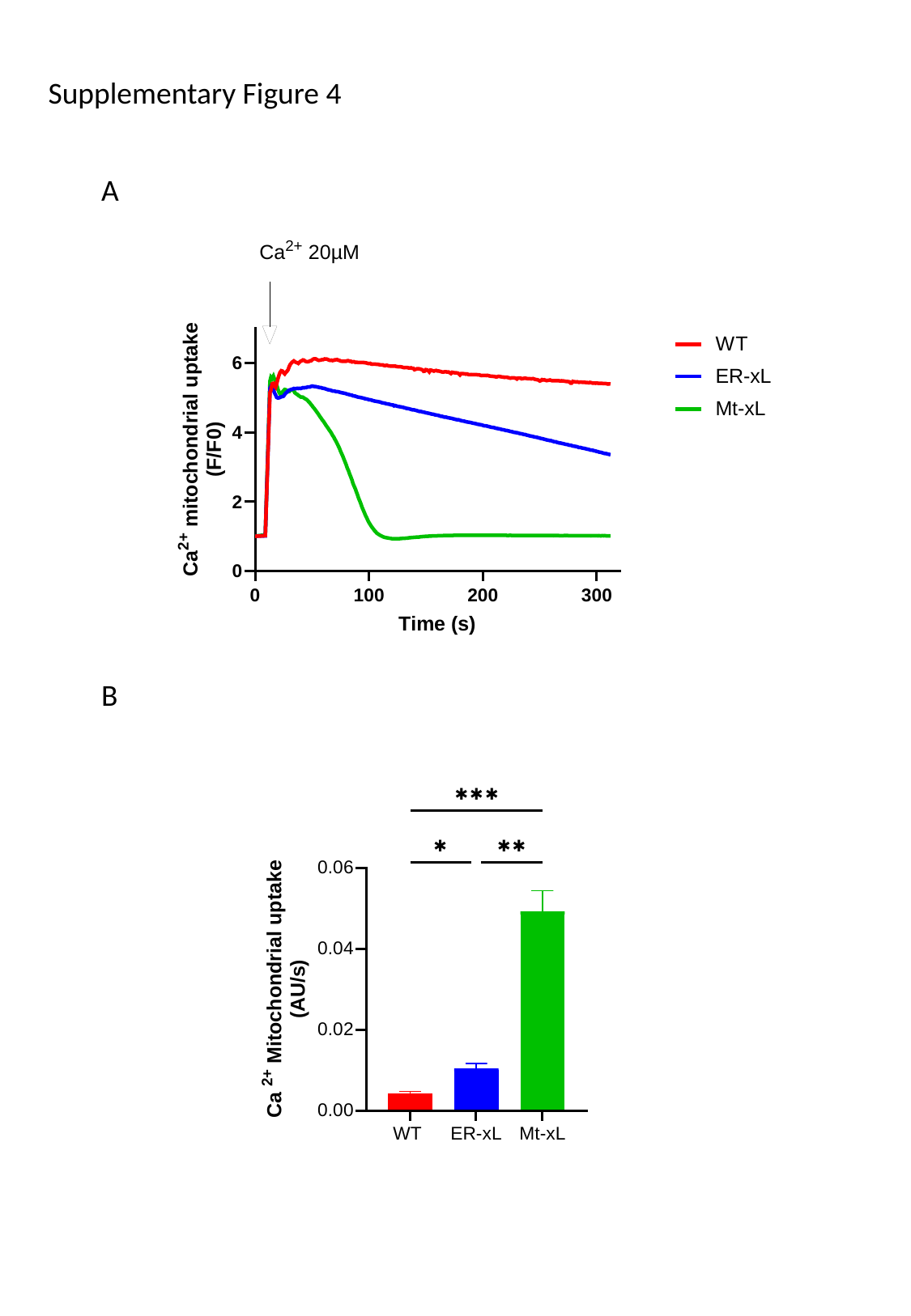

Supplementary Figure 4
A
B

## Slide 8
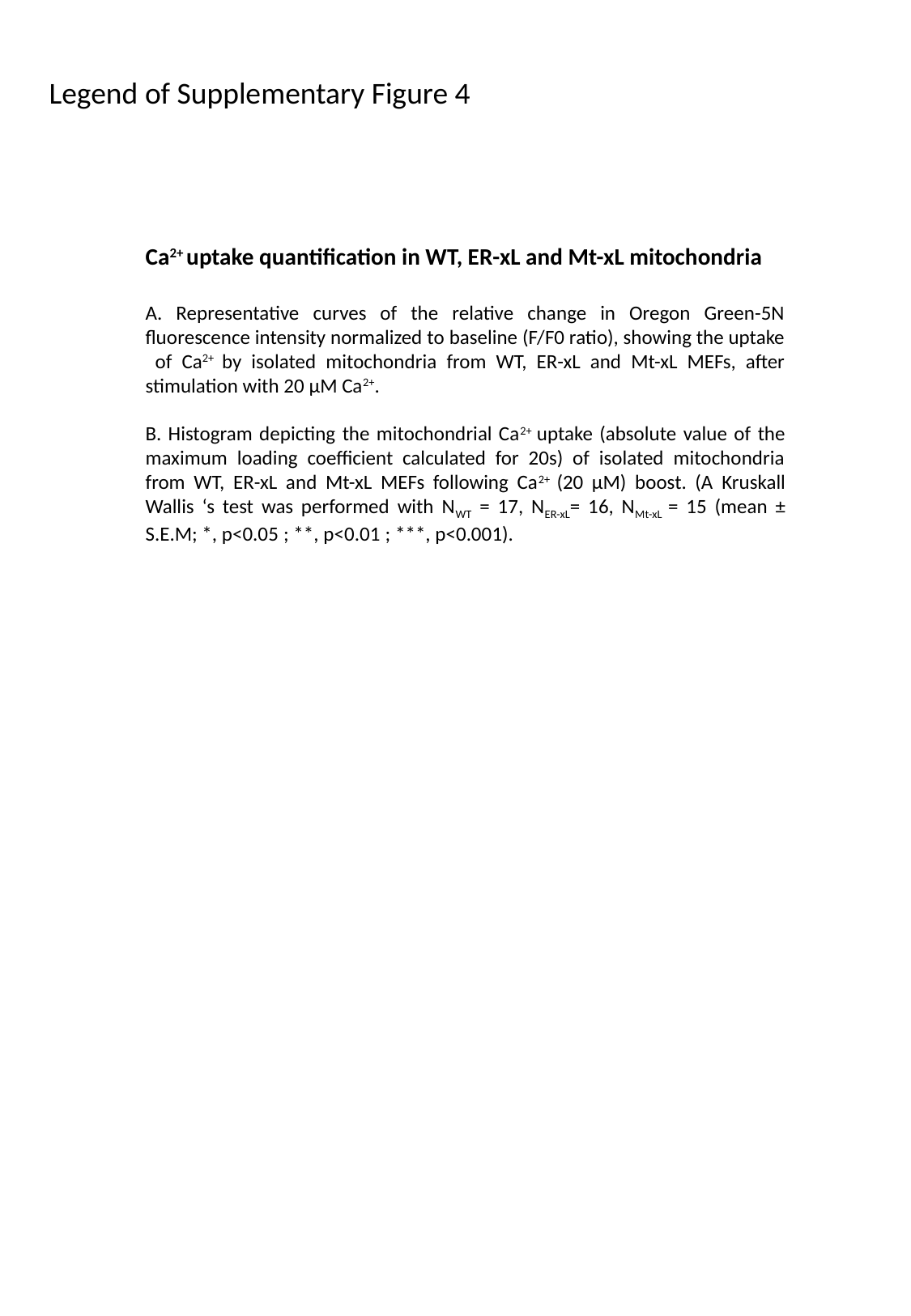

Legend of Supplementary Figure 4
Ca2+ uptake quantification in WT, ER-xL and Mt-xL mitochondria
A. Representative curves of the relative change in Oregon Green-5N fluorescence intensity normalized to baseline (F/F0 ratio), showing the uptake of Ca2+ by isolated mitochondria from WT, ER-xL and Mt-xL MEFs, after stimulation with 20 µM Ca2+.
B. Histogram depicting the mitochondrial Ca2+ uptake (absolute value of the maximum loading coefficient calculated for 20s) of isolated mitochondria from WT, ER-xL and Mt-xL MEFs following Ca2+ (20 µM) boost. (A Kruskall Wallis ‘s test was performed with NWT = 17, NER-xL= 16, NMt-xL = 15 (mean ± S.E.M; *, p<0.05 ; **, p<0.01 ; ***, p<0.001).

## Slide 9
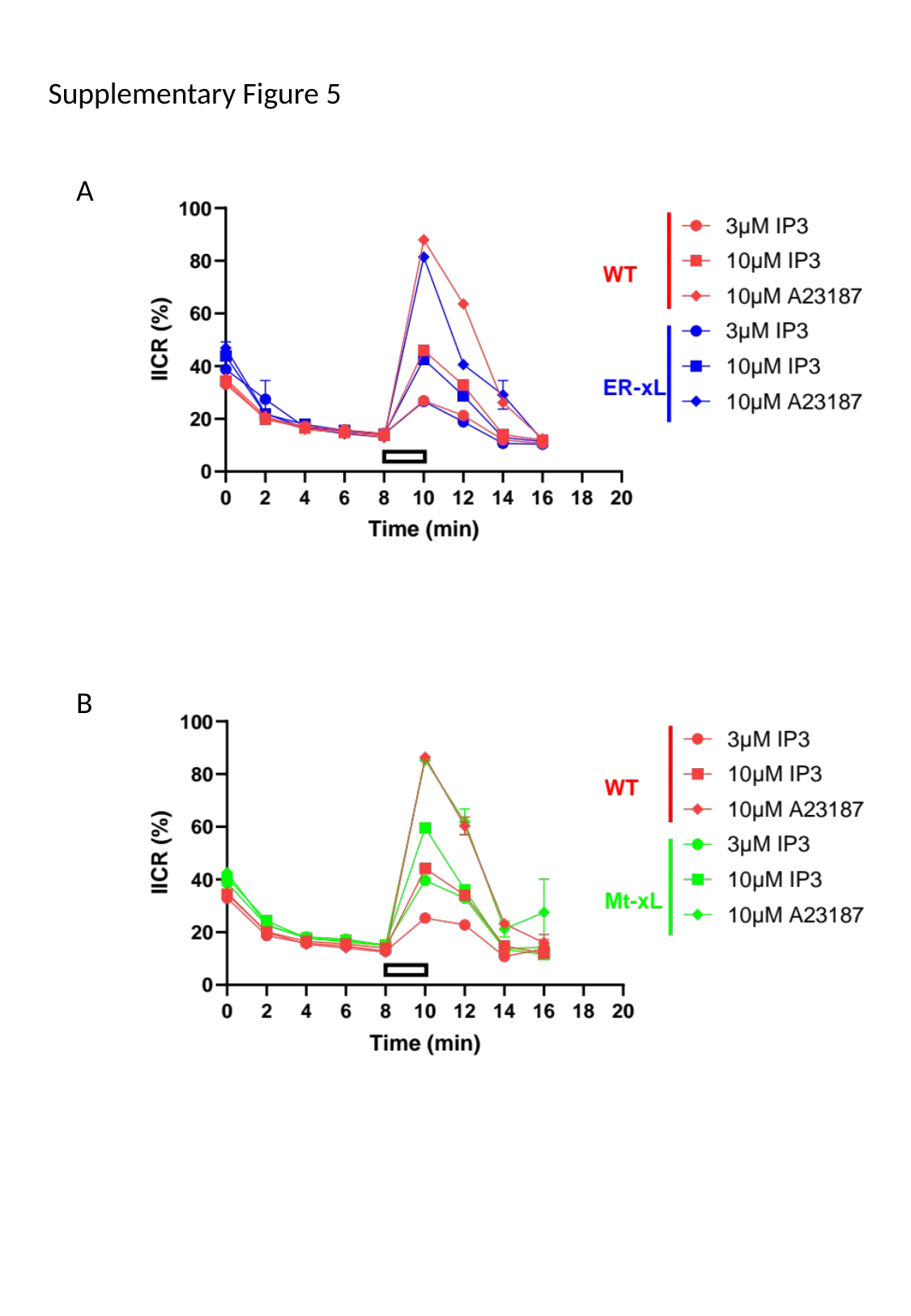

Supplementary Figure 5
A
B

## Slide 10
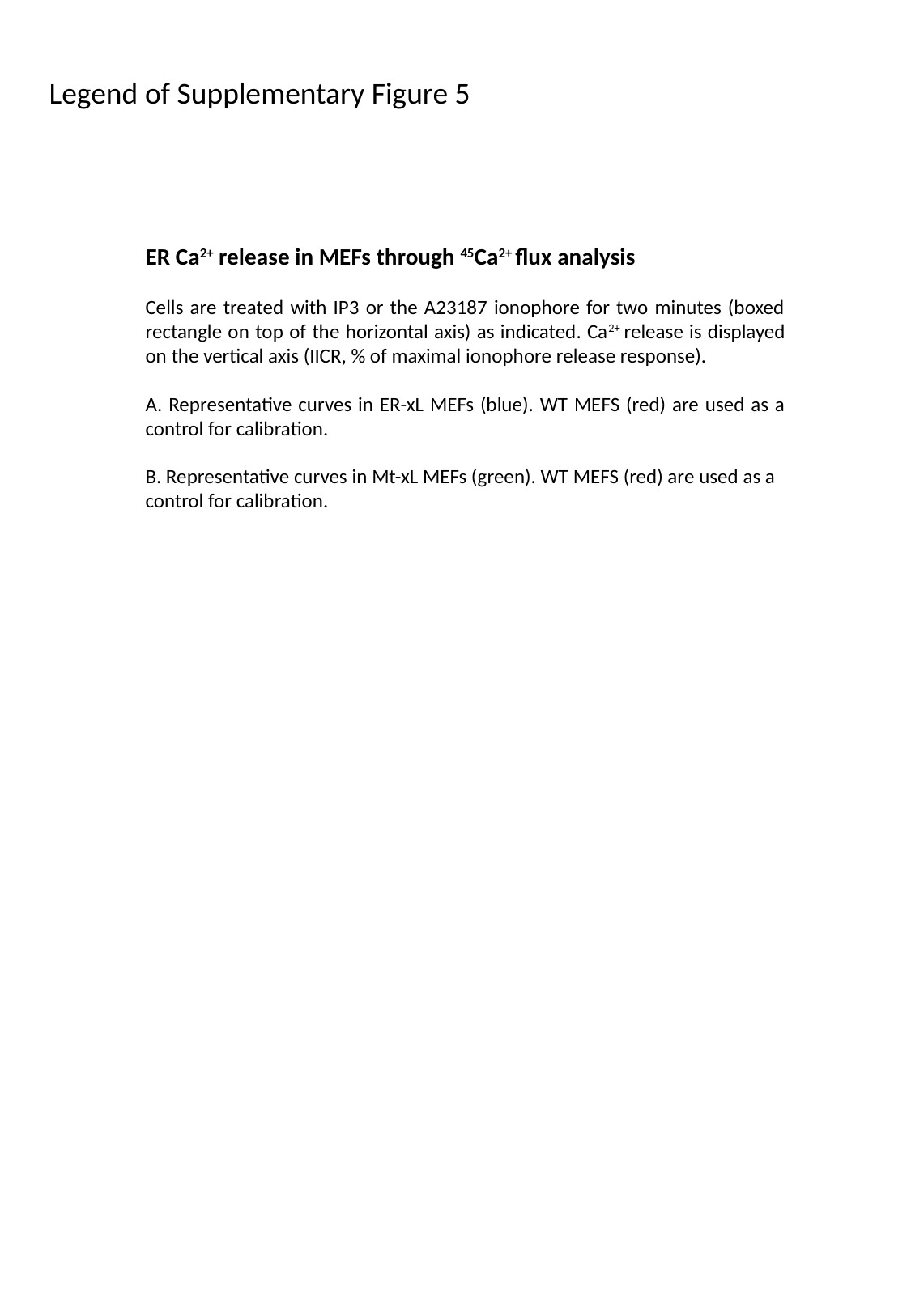

Legend of Supplementary Figure 5
ER Ca2+ release in MEFs through 45Ca2+ flux analysis
Cells are treated with IP3 or the A23187 ionophore for two minutes (boxed rectangle on top of the horizontal axis) as indicated. Ca2+ release is displayed on the vertical axis (IICR, % of maximal ionophore release response).
A. Representative curves in ER-xL MEFs (blue). WT MEFS (red) are used as a control for calibration.
B. Representative curves in Mt-xL MEFs (green). WT MEFS (red) are used as a control for calibration.
